# Evaluating the psychosocial effects of the Northern Territory Institute of Sport (NTIS) High Performance Officiating Program (HPOP)

**DOI:** 10.1371/journal.pone.0354971

**Published:** 2026-08-05

**Authors:** Elizabeth M. Grylls, Graham Glassford, Stuart Carrington, Martin J. Turner

**Affiliations:** 1 Charles Darwin University, Darwin, Australia; 2 Northern Territory Sports Academy, Darwin, Australia; 3 St Mary’s University, London, United Kingdom; 4 School of Psychology, Manchester Metropolitan University, Manchester, United Kingdom; Mugla Sitki Kocman University: Mugla Sitki Kocman Universitesi, TÜRKIYE

## Abstract

This study investigates the effects of the 2021 Northern Territory Institute of Sport (NTIS) High Performance Officiating Program (HPOP) on five officials. The HPOP is designed to develop and support officials to aspire to and achieve high-level and international standards of officiating [1] through specialised training, mentorship, and resources to enhance their skills and performance in high-level officiating. At pre- and post-program, data were collected from each official and their mentor pertaining to official self-efficacy and resilience. Data were also collected on challenge and threat appraisals, physical and mental preparation, locus of control, and functional assertiveness subscales. Officials and mentors were also asked qualitative questions on resilience and self-efficacy. Visual analyses of graphed data, Reliable Change Indices (RCIs), and social validation data indicated reliable improvements in self-efficacy and resilience for three of the five officials. Changes were also observed in self-efficacy subscales (game knowledge, decision-making, pressure, and communication) although patterns varied between officials. Similarly, challenge and threat appraisals, physical and mental preparation, locus of control, and functional assertiveness demonstrated individualised patterns of change. Qualitative findings provided further support for the program’s positive influence on self-efficacy and resilience. This research contributes valuable insights into the transformative effects of the NTIS High Performance Officiating Program on officials, emphasising notable improvements in self-efficacy and resilience.

## Introduction

Sports officials play a critical role in ensuring fair play, upholding the rules, and are required to maintain order in many environments, including outdoor (e.g., field, pitch, range, course) and indoor sporting venues (e.g., arena, swimming pool, court, bowling alley, ring). Similar to other sports performers, including athletes and coaches, sports officials are expected to perform their roles under pressure, make critical decisions, have effective communication, and be resilient in the face of criticism. Research in the area of sport psychology has focused predominantly on athletes and coaches with relatively little attention focused on the psychological needs and challenges that are faced by sporting officials (see Forbes & Livingston [1]; Grylls et al., 2021 [2]; Maxwell-Keys et al., 2022 [3], for some exceptions). In comparison to athletes, much less consideration has been given to identifying and supporting the psychological needs of officials, despite their critical role in the sport environment. As a result, structured training and development specific to sporting officials began to emerge in the early 2000s [4]. Research that seeks to understand how officials can be psychologically supported is needed because a significant number of sports officials drop out each year. A report by the Australian Bureau of Statistics [5] revealed a 26% decline in the number of sports officials in Australia, from 456,800–340,000 between 1997 and 2001. Recent data from the Australian Sports Commission [6] indicates 5% of the population in the non-playing role of official in 2016 to 4.7% in 2021, and 4.3% in 2024.

Currently, there are few opportunities for sports officials to undertake high performance training, compared to coach and athlete development [7]. Further, in 2021 the Northern Territory Institute of Sport (NTIS) was the only Institute or Academy of Sport in Australia to offer sports official scholarships for officials across multiple sports within an officiating program for a twelve month period, titled the NTIS High Performance Officiating Program (HPOP) [8]. The HPOP provided opportunities and encouraged officials from the Northern Territory to aspire to and achieve high level national and international standards of officiating. The HPOP aimed to develop each participating official holistically through developing their physical, psychological, nutritional, and game knowledge through a range of activities, including a “give-back” project, weekend workshops, education sessions, regular meetings between each official and mentor, and individual sessions with service providers. However, an aspect of the HPOP that is understudied is the psychological impact of the program on participating officials. Given the broad remit of the HPOP program, to evaluate it requires an equally broad approach. To this end, in the current study the researchers selected psychological markers that have been studied in previous literature concerning tennis officials [2], namely resilience, challenge and threat evaluation, and self-efficacy, to assess the psychological effects of the HPOP program on participating officials.

Understanding resilience across sporting officials may provide some insight into the retention of sporting officials. Resilience can be defined as “the process by which individuals are able to positively adapt to substantial difficulties, adversity, or hardship” [9, p. 592]. Due to the complexity and importance of the tasks officials perform, their role is usually associated with high levels of stress [10]. Stress and adversity are important in developing resilience and “without any there is nothing to be resilient against” [9, p. 586]. Stress among sports officials has been studied across various sports, including rugby union referees [e.g., 11], basketball referees [e.g., 12], officials from aesthetic sports, such as diving and gymnastics [e.g., 13], and handball referees [e.g., 14]. The research consistently shows that stress negatively affects performance, particularly in areas such as decision-making, concentration, and confidence [12]. While a moderate level of stress may enhance alertness, chronic or unmanaged stress is linked to burnout [11], reduced retention, and intentions to quit officiating [15].

Both stress and resilience are thought to be impacted upon by cognitive appraisal, and specifically, challenge and threat appraisals. Within cognitive appraisal frameworks, a challenge state is characterised by an individual’s confidence in their capacity to meet situational demands [16]. In contrast, a threat state reflects an appraisal in which perceived demands exceed available coping resources [17]. Turner and Barker [18] suggest that, in the face of adversity, greater resilience is demonstrated by displaying a challenge state, leading to positive (or less negative) outcomes during performance situations. Conversely, poorer resilience is indicated by exhibiting a threat state, resulting in negative (or less positive) outcomes during performance situations [16]. Turner and Barker draw on the theory of challenge and threat state in athletes [TCTSA; 19], to highlight that through stressful experiences one can better adapt to future stressors, an idea referred to as resilience by Seery [20]. Evidence from extant literature indicates that threat states, underpinned by threat appraisal, are associated with poorer performance while a challenge states, underpinned by challenge appraisal, are associated with superior performance [19,21,22]. Turner and Barker [18] also point out that self-efficacy is a key factor in determining challenge and threat states, with greater self-efficacy predictive of greater challenge [subsequently born out in research findings; [23, 24]. Indeed, such is the importance of self-efficacy, research has also found that even when athletes experience a threat state, higher self-efficacy is still predictive of superior athletic performance [25]. In officials, Guillén and Feltz [26] define referee self-efficacy as the extent to which referees believe they have the capacity to perform successfully.

In a study concerning self-efficacy, challenge and threat, and resilience with officials, Grylls et al. [2] found that self-efficacy, challenge appraisal, and resilience were positively related, whilst threat was negatively related to resilience. Although interesting and informative, the study by Grylls et al. [2] offers only a cross-sectional snapshot of official self-efficacy, challenge and threat appraisal, and resilience. Whether and to what extent programs such as HPOP serve to enhance these important psychological nutriments has not been tested in literature, and indeed, sparse research has reported interventions that can promote challenge appraisal (see Williams et al. [27, for an exception]). Given the importance of said constructs, it is likely that building self-efficacy, resilience, and challenge and threat appraisals in sporting officials may have a positive impact on the retention of sporting officials, enhance the officials’ experience of officiating, and officiating behaviours. For example, officials with high self-efficacy and resilience, are more likely to believe in their abilities and are more likely to stay involved in their sport [28]. Further, officials who appraise stressful situations as a challenge are more likely to derive enjoyment from them, while those who interpret these situations as a threat may experience heightened anxiety and stress [29]. Also, resilient officials with high self-efficacy are more likely to make more accurate and confident decisions [26].

Aside from the discussed factors, there are of course many other factors that can impact the performance of officials. For example, locus of control is the tendency of individuals to perceive the events impacting them positively or negatively as a result of their own behaviours or external factors [30]. Research has linked locus of control to decision-making in Kayseri football officials [31]. Another crucial behaviour for officiating is functional assertiveness, which can assist officials in making accurate decisions [32]. Although Wilson and Mock [32] found that assertiveness alone did not correlate with accuracy in making penalty calls for ice-hockey referees, when assertiveness was combined with higher levels of certification, those referees were the most accurate. As such, in the current study we also assess the impact of the HPOP on physical and mental preparation, locus of control, and functional assertiveness subscales.

To guide the assessment of HPOPs’ impact on key outcomes in officials, we drew on the Theory of Challenge and Threat States in Athletes (TCTSA; 19). In the TCTSA, and therefore the present study, how an individual adapts to stressful situations is thought to be governed by a transactional process of cognitive appraisal. In the TCTSA, one’s appraisal of situation demands and psychological resources shapes one of two outcome states; challenge or threat. Challenge is considered to be an indicator of psychological resilience (20). Challenge reflects sufficient resources to meet demands, whilst threat reflects insufficient resources to meet demands. In the TCTSA resources are self-efficacy, perceived control, and goal orientation, with greater self-efficacy, greater control, and a focus on approach goals rather than avoidance goals indicative of challenge. In evaluating the HPOP we attempted to align our measurement procedures with TCTSA, adopting psychometrics that were valid for use in the sampled population and tapping important outcomes for officials. There is no one single measure that can determine challenge and threat, so our approach mirrored previous research [e.g., 2, 23, 33] in developing a multi-measure procedure to evaluate the HPOP. Although the TCTSA was originally developed within athlete performance contexts, research has applied challenge-threat theory to non-athlete performers, including firefighters [34], police officers [35], and sports officials [2], supporting its relevance for understanding stress appraisal and performance in other high-pressure roles, such as sports officiating.

The present study represents the first empirical evaluation of the HPOP to our knowledge. The primary aim of the study is to examine the longitudinal changes in self-efficacy, resilience, challenge and threat appraisals, physical and mental preparation, locus of control, and selected psychological characteristics associated with officiating performance among officials engaged in the HPOP.

## Methods

### Participants

Six sporting officials applied for the 2021 NTIS’s High Performance Officiating Program (HPOP) intake and were subsequently selected. One official did not complete all requirements of the study (due to Covid19 restrictions), hence the study reports on five officials. The five officials had an average age of 44.6 years (*SD* = 16.56), with one male and five females involved in the study. The participants had been officiating for an average of nine years (*SD* = 11.94) with a range of one to 30 years and had officiated on average a total of 20.20 days (*SD* = 20.18) in the last twelve months. Two of the participants had officiated at club level, two at the State/Territory level, and one at a National level. Four of the officials aimed to officiate at a national level within the next five years and one aimed to officiate at the international level within the next five years. The official’s sports were Hockey, BMX, Golf, Touch Football, and Netball.

### HPOP Schedule

Following ethical approval from Charles Darwin University, an induction session was held in December 2020 to outline the HPOP program and its requirements. All participants in the 2021 HPOP attended a two-day workshop in February 2021 and a two-day workshop in November 2021. Between these two weekends, participants had access to a series of monthly non-compulsory workshops. These sessions featured elite officials from a range of sports (e.g., AFL, NRL, and World Athletics), who shared pathways to high-level officiating and focused on key topics such as communication and conflict management. In addition, service providers delivered workshops and information sessions on a variety of relevant themes.

Table 1 outlines the schedule of workshops and sessions delivered throughout the 2021 NTIS High Performance Officiating Program (HPOP). The HPOP is a government funded performance development initiative administered by the NTIS. The HPOP was an existing program designed and governed by the NTIS to support the development of emerging and high-performance sports officials in the Northern Territory. The program was not developed by the authors. The relatively small cohort size reflects the limited pool of officials operating at, or progressing towards, national-level competition within the Northern Territory performance pathway.

**Table 1:** HPOP Workshops for all officials and mentors from December 2020 – November 2021.

Date	Session Type	Brief Overview
December 2020	Induction	This provided information on the program, what the expectations were and the time commitment prior to signing the agreement.
February 2021	Introduction Workshop (two days)	All officials met with individual service providers (psychologist, nutritionist, physical preparation, mentor/mentee agreement discussion), workshop sessions on resilience, movement improvisation, learning styles (VARK), DISC Profiling, Communication, Goal Setting, the Mentoring Relationship, Nutrition, Reflective Practices
March 2021	Self-Regulation – Psychologist	Workshop on self-regulation, e.g., the ability to control one’s behaviour, thoughts, emotions that are directed towards specific goals
April 2021	NRL Referee Coach discussion	Discussion with NRL Referee Coach with a focus on communication between the official and athlete and how to create better connections.
May 2021	AFL Official discussion	Discussion with AFL Umpires. Group chat on pathway/journey of umpire/challenges/pre and post routines/ feedback from coaches
June 2021	DISC Profile – Session 2	Identify Officiating style is and the 3 Behavioural strengths that create this style. Identify 3 behavioural areas to develop. Identify a fellow Official whom they have to work with in their role. Explain what their profile is and how they would build a productive working relationship with them.
June 2021	HPOP manager check-in meeting with each official	Six-month review on how each official is tracking against their goals set at the start of the program.
July 2021	Conflict Management –AFL	Workshop on Conflict Resolution. Video examples and discussion on how to deal with conflict and how does their DISC profile work in each example
August 2021	Self-efficacy – Psychologist	Workshop on Self-efficacy. Discussion on ways to build confidence, how to ensure they are physically and mentally prepared, vicarious experiences, verbal persuasion, positive self-talk, imagery, and emotion control,
September 2021	The Olympic Experience – Athletics Australia	Discussion with Athletics Australia about the Olympic Experience and managing conflict. Spoke about real examples of dealing with stress situations as the Start Line Referee at the 2020 Tokyo Olympics.
October 2021	Unconscious Bias	Workshop on Unconscious Bias. Focus on cognitive bias in officiating. Information on how to recognise and activities to identify biases.
November 2021	End of Year Workshop (two days)	All officials met with individual service providers (psychologist, nutritionist, physical preparation, mentor/mentee), exit interviews conducted between officials and HPOP Manager, Shopping Tour with Nutritionist, Lessons Learnt (Psychology and Nutrition), each official presented to the group what they have learnt across the year (including their “Give Back” Project), session on leadership, HPOP Wrap up.

Notes: At each workshop the following items were implemented regardless of the session type:

• Discussion at the start of each workshop around successes officials had and any challenges from the previous month.

• From the previous session, did the official implement something that they learnt from the last session.

• At the end of the workshop there was a deliberate reflective practice on what each official learnt from the session.

Within this program, the lead author was engaged by the NTIS in an applied consultancy role to plan and facilitate the psychology-focused components of the curriculum. Specifically, the resilience session introduced officials to key psychological foundations of resilience and equipped them with practical strategies to manage stress and pressure, including challenge appraisal and the influence of personal and environmental factors on resilience development. The self-regulation workshop focused on enhancing officials’ capacity to manage thoughts, emotions, and behaviours in pursuit of performance goals, while the self-efficacy workshop targeted officials’ confidence in their ability to perform effectively under pressure.

Beyond these dedicated psychological sessions, psychological skills development was integrated into several broad program workshops designed and delivered by NTIS and other content specialists. The February two-day introductory workshop included sessions on communication, goal setting, reflective practice, and DISC profiling (a behavioural framework categorising Dominance, Influence, Steadiness, and Conscientiousness types to enhance self-awareness), all of which contribute to psychological preparedness. Later workshops on conflict management (July) and unconscious bias (October) further supported psychological development by targeting interpersonal skills, decision-making, and self-awareness under pressure. The final two-day workshop in November reinforced psychological learning through reflective presentations, exit interviews, and structured discussions on lessons learnt, including psychological and nutritional insights. Officials also presented their “Give Back” projects, providing opportunities for guided self-reflection, consolidation of learning, and confidence-building.

Due to Covid-19 restrictions, a number of the sessions were conducted online only, likewise, if an official was unable to attend due to Covid-19, they were able to attend remotely. Along with attending the regular sessions, each official in the program also had a mentor whom they were encouraged to regularly meet through a formal mentoring approach whereby both parties set out timing of meetings and goals. Each official was also required to develop a “Give-Back” project to their sport.

### Design

We took a longitudinal repeated-measures approach to the study, whereby officials completed measures that assessed the target variables at four timepoints over a period of ten months (on commencement of the program, twice during the program, and on completion of the program). We also adopted an idiographic approach to quantitative data analyses and presentation, whereby each participants’ data are analysed and presented individually, in order to facilitate the individualised analysis of intervention effects whereby more can be learned by studying fewer participants [36]. This is because each participant should be seen as an individual whose journey through the HPOP is particular to them, and also because amalgamating the data would lose nuance that could be instructive for future researchers and practitioners. This approach is in line with recent applied research [e.g., 37, 38] and contributes to the increase of idiographic research approaches in sport and exercise psychology [39]. This method is encouraged by scholars to seek improvements in scientific understanding to inform applied practice [40,41]. In addition to quantitative data, several open questions were asked of the officials and their mentors, specifically around changes and development in self-efficacy and resilience. The mentors were asked to rate the self-efficacy of their official (via a quantitative questionnaire) and briefly discuss the change and development of their official regarding self-efficacy and resilience.

All officials and mentors completed a consent form after reading a plain language statement before any data was collected. The data was collected through a paper-based questionnaire, with each official and mentor encouraged to complete it within two weeks of receiving the pack. Data was collected at four-time points: baseline (time 1), twice during the program (time 2, and time 3), and on program completion (time 4). Data collection occurred in February (time 1), May (time 2), August (time 3), and November (time 4).

At time 1, officials completed a demographic questionnaire, and measures of resilience, self-efficacy (including physical or mental preparation), challenge and threat appraisal, locus of control, and assertiveness scales. At time 2, 3 and 4, officials completed the same psychological measures, excluding the demographic information, and qualitative questions exploring changes in resilience and self-efficacy. The qualitative questions asked were: “How has your self-efficacy changed since commencing the HPOP?”, “Please give an example of how you have shown increased self-efficacy since commencing in the HPOP?”, “How has your resilience developed since the commencement of the HPOP?”, and “Please give an example of how you have shown increased resilience since commencing in the HPOP?”,

At times 2, 3 and 4, mentors completed a quantitative measure of the official’s self-efficacy and responded to qualitative questions on resilience and self-efficacy. The questions were “How has your official’s self-efficacy changed since commencing the HPOP?”, “Please give an example of how your official has shown increased self-efficacy since commencing in the HPOP?”, “How has your official’s resilience developed since the commencement of the HPOP?”, and “Please give an example of how your official has shown increased resilience since commencing in the HPOP?”

### Measures

**Demographic information.** Participants completed a number of questions including age, gender, what their primary role was in officiating, if they officiate any other sports, the number of years that they have officiated in their sport, played and/or coached their sport that they officiate in. They were also asked approximately how many tournaments/matches and how many days they had officiated in the past 12 months.

**Resilience.** The Connor-Davidson Resilience Scale-10 (CD-RISC10: [42]) is a self-rated measure of resilience and adaptability [43]. The instrument requires responses to ten statements (e.g., “I am able to adapt to change”) using a five-point Likert-scale ranging from 1 (*Not true at all*) to 5 (*True nearly all the time*) which are used to calculate a single overall score through summing the score on each item. The CD-RISC10 has been shown to be valid and to have high internal reliability [44], and has been used with officials [e.g., 2].

**Challenge and Threat Appraisals.** Challenge and threat appraisals were measured using the Cognitive Appraisal Scale [CAS; 45]. The CAS is an 18-item Likert-type scale in which item responses range from 1 (*strongly disagree*) to 6 (*strongly agree*) and participants were asked to indicate the extent to which they agreed with each statement. Eight items make up the Challenge subscale (e.g., “I tend to focus on the positive aspects of any situation”). Ten items make up the Threat subscale (e.g., “I am concerned that others will find fault with me”). The CAS is used widely in challenge and threat research in athletic and work contexts [e.g., 23].

**Self-efficacy.** Referee Self-Efficacy Scale (REFS) measures the extent to which a referee believes that they have the ability to successfully officiate a competition [46]. The REFS consists of 13 items, and the stem for all items is “in the context of performing your officiating role, how confident are you in your ability to…”. Each item relates to one or more factors of referee self-efficacy: game knowledge (confidence in knowledge of their sport, including rules, officiating mechanics, and basic game strategy), decision making (confidence that referees have in their ability to quickly and firmly make decisions during competition), pressure (confidence that referees have in their ability to be uninfluenced by pressure from players, spectators, and coaches), and communication (confidence that referees have in their ability to communicate effectively with other referees, coaches, players, and auxiliary personnel). The Total REFS was calculated by summing all 13 items. Higher scores indicate greater self-efficacy. Previous research [e.g., 47, 48] has found the REFS to be valid and reliable. Myers et al. [46] showed support for the sources of referee self-efficacy as significant predictors of the four dimensions of REFS.

**Physical and Mental Preparation from the Sources of self-efficacy.** The Sports Officials Self-Rating Scale [26] was used to evaluate sources of self-efficacy. In the current study, based on previous research [e.g., 2], officials were asked to rate their physical and mental preparation across 5 items on a 7-point scale from 1 (*Not at all important*) to 7 (*Of highest importance*). The stem for all items is “I gain confidence in officiating when I….”

**Locus of Control.** Rotter’s Locus of Control Scale [49] is a 29-item questionnaire that measures the degree to which the individual interprets events as being a result of their own actions or external factors. Those with an ‘internal locus of control’ believe they can exercise control over events in their life, and that outcomes are determined by their own effort and abilities. Those with an ‘external locus of control’ do not believe their behaviour or decision making has much impact, but rather that things are decided by external forces such as fate, chance, or powerful others. Scores range from 0 to 23, with lower scores indicating internal control and higher scores indicating an external locus of control.

**Functional Assertiveness Scale.** The Functional Assertiveness Scale assesses the interpersonal communication between parties [50], and assesses an individual’s ability to express themselves effectively and appropriately, balancing objective effectiveness with pragmatic politeness [51]. The 12-item measure asks respondents how satisfied they would be with the outcomes of specific interpersonal situations, such as wanting someone to do something or to stop doing something. Each item is scored on a five-point scale between 1 (*always disagree*) and 5 (*always agree*). The scale comprises two subscales: Objective Effectiveness, which assesses the extent to which an individual achieves their intended outcome in an interpersonal interaction, and Pragmatic Politeness, which assesses the extent to which interpersonal interactions are conducted in a socially appropriate and considerate manner [51].

### Data analysis – quantitative data

Three indicators were used to determine the effects of the HPOP program across the four timepoints: percentage (%) change, Mean change, and reliable change. Similar to other applied sport psychology literature [e.g., 52], the Reliable Change Index [RCI; 53] was used to indicate whether the change in the official’s scores over time were significant and not due to measurement error or natural fluctuations. RCI is calculated by dividing the difference between the pre- and post-test scores by the standard error of the difference (SE_D_) between the two scores [53]. In the current study, this was done from baseline (time 1) to time 2, time 2–3, and baseline (time 1) to time 4. An RCI value greater than 1.96 or less than −1.96 indicates a significant change at the 95% confidence level. All RCIs across all participants and variables can be located in Tables 2–6 of supplementary materials. To aid brevity in reporting the results, intext we focus on changes between baseline and Time 4 (i.e., the study period) as shown in Tables 7 and 8.

**Table 2:** Mean scores, percentage (%) change, Mean change, and RCI for Resilience, Self-Efficacy (SR), and Self-Efficacy (MR) across baseline, time 2, time 3, and time 4. Change data (M, %, RCI) reflect change from the previous timepoint, apart from the final column which reflects change from baseline to time 4. N.B. ^a^Baseline to time 2 mean % change, ^b^Time 2 to time 3 mean % change, ^c^Time 3 to time 4 mean % change, ^d^baseline to time 4 mean % change.

Measure	Official	Baseline	Time 2				Time 3				Time 4				Baseline to Time 4		
			*M Score*	%	*M Change*	*RCI*	*M Score*	%	*M Change*	*RCI*	*M Score*	%	*M Change*	*RCI*	%	*M Change*	*RCI*
Resilience	Jamie	24	26	8.33^a^	2	1.04	33	26.92^b^	7	**2.72**	33	0.00^c^	0	0.00	37.50^d^	9	**3.99**
	Alex	24	31	29.17^a^	7	**3.65**	33	6.45^b^	2	0.68	33	0.00^c^	0	0.00	37.50^d^	9	**3.99**
	Meg	30	31	3.33^a^	1	0.52	31	0.00^b^	0	0.00	34	9.68^c^	3	0.95	13.33^d^	4	**1.77**
	Jade	26	24	−7.69^a^	−2	−1.04	25	4.17^b^	1	0.34	33	32.00^c^	8	**2.53**	34.62^d^	7	**3.11**
	Kate	22	26	18.18^a^	4	**2.08**	20	−23.08^b^	−6	**−2.04**	24	20.00^c^	4	1.26	9.09^d^	2	0.89
Self-Efficacy (SR)	Jamie	57	62	8.77^a^	5	1.35	59	−4.83^b^	−3	−0.81	55	−6.78^c^	−4	−1.79	−3.51^d^	−2	−0.89
	Alex	44	44	0.00^a^	0	0.00	52	18.18^b^	8	**2.16**	53	1.92^c^	1	0.45	20.45^d^	9	**4.00**
	Meg	53	61	15.07^a^	8	**2.15**	58	−4.92^b^	−3	0.81	62	6.90^c^	4	1.79	16.98^d^	9	**4.00**
	Jade	54	58	7.41^a^	4	1.08	63	8.62^b^	5	1.35	57	−9.52^c^	−6	**−2.69**	5.56^d^	3	1.33
	Kate	50	55	10.00^a^	5	1.35	56	1.82^b^	1	0.27	57	1.79^c^	1	0.45	14.00^d^	7	**3.11**
Self-Efficacy (MR)	Jamie	44	49	11.36^a^	5	1.76	48	−2.04^b^	−1	−0.34	48	0.00^c^	0	0.00	11.36^d^	4	1.44
	Alex	37	57	81.08^a^	20	**7.06**	61	7.02^b^	4	1.35	62	1.64^c^	1	0.28	67.57^d^	25	**7.20**
	Meg	50	56	10.00^a^	5	1.76	64	14.29^b^	8	**2.70**	63	−1.56^c^	−1	−0.28	26.00^d^	13	**3.74**
	Jade	47	55	17.02^a^	8	**2.82**	54	−1.82^b^	−1	−0.34	56	3.70^c^	2	0.56	19.15^d^	9	**3.47**
	Kate	47	49	4.26^a^	2	0.71	53	8.16^b^	4	1.35	49	−7.55^c^	−4	−1.12	4.26^d^	2	0.58

Significant results highlighted in bold**.**

**Table 3:** Mean scores, percentage (%) change, Mean change, and RCI for Challenge Appraisal, Threat Appraisal and Physical and Mental Preparation across baseline, time 2, time 3, and time 4. Change data (M, %, RCI) reflect change from the previous timepoint, apart from the final three columns which reflects change from baseline to time 4. N.B. ^a^Baseline to time 2 mean % change, ^b^Time 2 to time 3 mean % change, ^c^Time 3 to time 4 mean % change, ^d^baseline to time 4 mean % change.

Measure	Official	Baseline	Time 2				Time 3				Time 4				Baseline to Time 4		
			*M Score*	%	*M Change*	*RCI*	*M Score*	%	*M Change*	*RCI*	*M Score*	%	*M Change*	*RCI*	%	*M Change*	*RCI*
Challenge Appraisal	Jamie	41	37	−9.76^a^	−4	−1.22	39	5.41^b^	2	0.72	42	7.69^c^	3	1.12	2.44^d^	1	0.34
	Alex	31	38	22.58^a^	7	**2.14**	39	2.63^b^	1	0.36	38	−2.56^c^	−1	−0.37	22.58^d^	7	**2.38**
	Meg	39	41	6.45^a^	2	0.61	38	−7.32^b^	−3	−1.08	43	13.16^c^	5	1.87	10.26^d^	4	1.36
	Jade	31	30	3.23^a^	−1	−0.31	35	16.13^b^	5	1.80	41	17.14^c^	6	**2.24**	32.26^d^	10	**3.40**
	Kate	32	35	9.38^a^	3	0.92	30	−14.29^b^	−5	−1.80	34	13.33^c^	4	1.49	6.25^d^	2	0.68
Threat Appraisal	Jamie	38	21	44.74^a^	−17	**−4.09**	19	−9.52	−2	−0.34	16	15.79^c^	−3	−0.39	−57.89^d^	−22	**−3.33**
	Alex	27	30	11.11^a^	3	0.72	23	−23.33^b^	−7	−1.19	28	21.74^c^	5	0.65	3.70^d^	1	0.15
	Meg	40	35	12.50^a^	−5	−1.20	39	11.43^b^	4	0.68	40	2.56^c^	1	0.13	0.00^d^	0	0.00
	Jade	30	30	0.00^a^	0	0.00	20	−33.33^b^	−10	−1.70	15	25.00^c^	−5	−0.65	−50.00^d^	−15	**−2.27**
	Kate	42	41	2.38^a^	−1	−0.24	40	−2.44^b^	−1	−0.17	45	12.5^c^	5	0.65	7.14^d^	3	0.45
Physical and Mental Preparation	Jamie	30	35	16.67^a^	5	**2.22**	29	−17.14^b^	−6	**−2.66**	30	3.45^c^	1	0.54	0.00^d^	0	0.00
	Alex	32	33	3.13^a^	1	0.44	31	−6.06^b^	−2	−0.89	34	9.68^c^	3	1.61	6.25^d^	2	1.08
	Meg	26	30	15.38^a^	4	1.78	30	0.00^b^	0	0.00	27	10.00^c^	−3	−1.61	3.85^d^	1	0.54
	Jade	28	27	−3.57^a^	−1	−0.44	25	−7.41^b^	−2	−0.89	29	16.00^c^	4	**2.15**	3.57^d^	1	0.54
	Kate	25	24	−4.00^a^	−1	−0.44	27	12.50^b^	3	1.33	25	7.41^c^	−2	−1.08	0.00^d^	0	0.00

Significant results highlighted in bold.

**Table 4:** Mean scores, percentage (%) change, Mean change, and RCI for Game Knowledge and Decision Making across baseline, time 2, time 3, and time 4. Change data (M, %, RCI) reflect change from the previous timepoint, apart from the final three columns which reflects change from baseline to time 4. N.B. ^a^Baseline to time 2 mean % change, ^b^Time 2 to time 3 mean % change, ^c^Time 3 to time 4 mean % change, ^d^baseline to time 4 mean % change.

Measure	Official	Baseline	Time 2				Time 3				Time 4				Baseline to Time 4		
			*M Score*	%	*M Change*	*RCI*	*M Score*	%	*M Change*	*RCI*	*M Score*	%	*M Change*	*RCI*	%	*M Change*	*RCI*
Game Knowledge (SR)	Jamie	13	13	0.00^a^	0	0.00	13	0.00^b^	0	0.00	14	7.69 ^c^	1	**2.13**	7.69^d^	1	1.73
	Alex	12	10	−16.67^a^	−2	**−2.41**	13	30.00^b^	3	**3.81**	13	0.00^c^	0	0.00	8.33^d^	1	1.73
	Meg	14	14	0.00^a^	0	0.00	14	0.00^b^	0	0.00	14	0.00^c^	0	0.00	0.00^d^	0	0.00
	Jade	11	13	18.18^a^	2	**2.41**	15	15.38^b^	2	**2.54**	13	−13.33^c^	−2	**−4.26**	18.18^d^	2	**3.46**
	Kate	12	12	0.00^a^	0	0.00	13	8.33 ^b^	1	1.27	13	0.00^c^	0	0.00	8.33^d^	1	1.73
Game Knowledge (MR)	Jamie	11	12	9.09^a^	1	0.90	12	0.00^b^	0	0.00	12	0.00^c^	0	0.00	9.09^d^	1	0.83
	Alex	10	14	40.00^a^	4	**3.60**	14	0.00^b^	0	0.00	15	7.14^c^	1	0.99	50.00^d^	5	**4.15**
	Meg	14	15	7.14^a^	1	0.90	15	0.00^b^	0	0.00	15	0.00^c^	0	0.00	7.14^d^	1	0.83
	Jade	12	14	16.67^a^	2	1.80	14	0.00^b^	0	0.00	15	7.14^c^	1	0.99	25.00^d^	3	**2.49**
	Kate	9	10	11.11^a^	1	0.90	12	20.00^b^	2	**2.23**	11	−8.33^c^	−1	−0.99	22.22^d^	2	1.66
Decision Making (SR)	Jamie	13	14	7.69^a^	1	0.85	14	0.00^b^	0	0.00	12	14.29^c^	−2	**−2.46**	−7.69^d^	−1	−1.38
	Alex	10	9	−10.00^a^	−1	−0.85	12	33.33^b^	3	2.43	12	0.00^c^	0	0.00	20.00^d^	2	**2.75**
	Meg	13	15	15.38^a^	2	1.70	14	−6.67^b^	−1	**−0.81**	15	7.14^c^	1	1.23	3.08^d^	2	**2.75**
	Jade	12	13	8.33^a^	1	0.85	15	15.38^b^	2	1.62	13	−13.33^c^	−2	**−2.46**	8.33^d^	1	1.38
	Kate	11	11	0.00^a^	0	0.00	12	9.09^b^	1	0.81	13	8.33^c^	1	1.23	18.18^d^	2	**2.75**
Decision Making (MR)	Jamie	10	11	10.00^a^	1	1.07	10	−9.09^b^	−1	−1.04	10	0.00^c^	0	0.00	0.00^d^	0	0.00
	Alex	7	11	57.14^a^	4	**4.29**	13	18.18^b^	2	**2.08**	13	0.00^c^	0	0.00	85.71^d^	6	**6.15**
	Meg	12	11	8.33^a^	−1	−1.07	15	36.36^b^	4	**4.17**	14	6.67^c^	−1	−1.00	16.67^d^	2	**2.05**
	Jade	10	12	20.00^a^	2	**2.15**	13	8.33^b^	1	1.04	15	15.38^c^	2	**2.00**	30.00^d^	3	**3.08**
	Kate	14	14	0.00^a^	0	0.00	11	−24.43^b^	−3	**−3.12**	12	9.09^c^	1	1.00	14.29^d^	−2	**−2.05**

SR = Self-Rated, MR = Mentor-Rated.

Significant results highlighted in bold.

**Table 5:** Mean scores, percentage (%) change, Mean change, and RCI for Pressure and Communication across baseline, time 2, time 3, and time 4. Change data (M, %, RCI) reflect change from the previous timepoint, apart from the final three columns which reflects change from baseline to time 4. N.B. ^a^Baseline to time 2 mean % change, ^b^Time 2 to time 3 mean % change, ^c^Time 3 to time 4 mean % change, ^d^baseline to time 4 mean % change.

Measure	Official	Baseline	Time 2				Time 3				Time 4				Baseline to Time 4		
			*M Score*	%	*M Change*	*RCI*	*M Score*	%	*M Change*	*RCI*	*M Score*	%	*M Change*	*RCI*	%	*M Change*	*RCI*
Pressure (SR)	Jamie	15	15	0.00^a^	0	0.00	15	0.00^b^	0	0.00	12	−20.00^c^	−3	**−3.20**	−20.00^d^	−3	**−2.27**
	Alex	9	10	11.11^a^	1	0.70	11	10.00^b^	1	0.85	12	9.09^c^	1	1.07	33.33^d^	3	**2.27**
	Meg	14	15	7.14^a^	1	0.70	15	0.00^b^	0	0.00	15	0.00^c^	0	0.00	7.14^d^	1	0.76
	Jade	15	14	−6.67^a^	−1	−0.70	14	0.00^b^	0	0.00	13	7.14^c^	1	1.07	−13.33^d^	−2	−1.51
	Kate	11	14	27.27^a^	3	**2.10**	14	0.00^b^	0	0.00	14	0.00^c^	0	0.00	27.27^d^	3	**2.27**
Pressure (MR)	Jamie	11	12	9.09^a^	1	0.59	12	0.00^b^	0	0.00	11	−8.33	−1	−0.60	0.00^d^	0	0.00
	Alex	8	13	62.50^a^	5	**2.94**	14	7.69^b^	1	0.55	14	0.00^c^	0	0.00	75.00^d^	6	**3.95**
	Meg	12	3	−75.00^a^	−9	**−5.30**	15	25.00^b^	12	**6.58**	15	0.00^c^	0	0.00	25.00^d^	3	**1.97**
	Jade	9	12	33.33^a^	3	1.77	10	16.67^b^	−2	−1.10	12	20.00^c^	2	1.21	33.33^d^	3	**1.97**
	Kate	10	10	0.00^a^	0	0.00	12	20.00^b^	2	1.10	11	−8.33^c^	−1	−0.60	10.00^d^	1	0.66
Communication (SR)	Jamie	16	20	25.00^a^	4	**3.47**	17	−15.00^b^	−3	**−2.86**	17	0.00^c^	0	0.00	18.75^d^	1	1.05
	Alex	13	15	15.38^a^	2	1.74	16	6.67^b^	1	0.95	16	0.00^c^	0	0.00	23.08^d^	3	**3.16**
	Meg	12	17	41.67^a^	5	**4.34**	15	11.76^b^	−2	−1.91	18	20.00^c^	3	**4.08**	50.00^d^	6	**6.32**
	Jade	16	18	12.50^a^	2	1.74	19	5.56^b^	1	0.95	18	5.26^c^	−1	−1.36	11.11^d^	2	**2.11**
	Kate	16	18	12.50^a^	2	1.74	17	−5.56^b^	−1	−0.95	17	0.00^c^	0	0.00	6.25^d^	1	1.05
Communication (MR)	Jamie	12	14	16.67^a^	2	1.66	14	0.00^b^	0	0.00	15	7.14^c^	1	0.75	25.00^d^	3	**2.19**
	Alex	12	19	58.33^a^	7	**5.80**	20	5.26^b^	1	0.86	20	0.00^c^	0	0.00	66.67^d^	8	**5.85**
	Meg	12	16	33.33^a^	4	**3.32**	19	18.75^b^	3	**2.57**	19	0.00^c^	0	0.00	58.33^d^	7	**5.12**
	Jade	16	17	6.25^a^	1	0.83	17	0.00^b^	0	0.00	17	0.00^c^	0	0.00	6.25^d^	1	0.73
	Kate	14	15	7.14^a^	1	0.83	18	20.00^b^	3	2.57	15	−16.67^c^	−3	**−2.25**	7.14^d^	1	0.73

SR = Self-Rated, MR = Mentor-Rated.

Significant results highlighted in bold.

**Table 6:** Mean scores, percentage (%) change, Mean change, and RCI for Locus of Control, Functional Assertiveness, Objective Effectiveness, and Pragmatic Politeness across baseline, time 2, time 3, and time 4. Change data (M, %, RCI) reflect change from the previous timepoint, apart from the final three columns which reflects change from baseline to time 4. N.B. ^a^Baseline to time 2 mean % change, ^b^Time 2 to time 3 mean % change, ^c^Time 3 to time 4 mean % change, ^d^baseline to time 4 mean % change.

Measure	Official	Baseline	Time 2				Time 3				Time 4				Baseline to Time 4		
			*M Score*	%	*M Change*	*RCI*	*M Score*	%	*M Change*	*RCI*	*M Score*	%	*M Change*	*RCI*	%	*M Change*	*RCI*
Locus of Control	Jamie	11	13	18.18^a^	2	1.19	10	−23.08^b^	−3	−0.99	9	−10.00^c^	−1	−0.52	−18.18^d^	−2	−1.04
	Alex	7	5	−28.57^a^	−2	−1.19	4	−20.00^b^	−1	−0.33	4	0.00^c^	0	0.00	−42.86^d^	−3	−1.56
	Meg	7	6	14.29^a^	−1	−0.60	7	16.67^b^	1	0.33	7	0.00^c^	0	0.00	0.00^d^	0	0.00
	Jade	9	10	11.11^a^	1	0.60	10	0.00^b^	0	0.00	10	0.00^c^	0	0.00	11.11^d^	1	0.52
	Kate	10	14	40.00^a^	4	**2.38**	20	42.86^b^	6	**1.99**	15	25.00^c^	−5	**−2.60**	50.00^d^	5	**2.60**
Objective Effectiveness	Jamie	20	21	5.00^a^	1	−0.41	23	9.52^b^	2	1.92	20	−13.04^c^	−3	−1.50	0.00^d^	0	0.00
	Alex	13	15	15.38^a^	2	0.82	19	26.67^b^	4	**2.09**	19	0.00^c^	0	0.00	15.38^d^	2	1.00
	Meg	18	18	0.00^a^	0	0.00	18	0.00^b^	0	0.00	21	16.67^c^	3	1.50	16.67^d^	3	1.50
	Jade	23	16	−30.43^a^	−7	**−2.88**	19	18.75^b^	3	1.56	19	0.00^c^	0	0.00	−17.39^d^	−4	**−1.99**
	Kate	24	24	0.00^a^	0	0.00	22	−8.33^b^	−2	−1.92	24	9.09^c^	2	1.00	0.00^d^	0	0.00
Pragmatic Politeness	Jamie	23	22	4.35^a^	−1	−0.51	26	18.18^b^	4	**2.17**	24	7.69^c^	−2	−1.26	4.35^d^	1	0.63
	Alex	18	18	0.00^a^	0	0.00	22	22.22^b^	4	**2.17**	23	4.55^c^	1	0.63	27.78^d^	5	**3.16**
	Meg	25	26	4.00^a^	1	0.51	26	0.00^b^	0	0.00	27	3.85^c^	1	0.63	8.00^d^	2	1.26
	Jade	23	22	−4.35^a^	−1	−0.51	27	22.73^b^	5	**2.71**	29	7.41^c^	2	1.26	26.09^d^	6	**3.79**
	Kate	24	27	12.50^a^	3	1.53	24	−11.11^b^	−3	−1.63	24	0.00^c^	0	0.00	0.00^d^	0	0.00

Significant results highlighted in bold.

**Table 7:** Participant Progress across Psychological Constructs over Time.

Participant	Resilience*				SE (SR)*				SE (MR)*				Challenge Appraisal*				Threat Appraisal**				PMP*			
	1	2	3	4	1	2	3	4	1	2	3	4	1	2	3	4	1	2	3	4	1	2	3	4
Jamie		**↑**	**↑**	**–**		↑	↓	↓		↑	↓	–		↓	↑	↑		**↓**	**↓**	**↓**		↑	↓	↑
Alex		**↑**	**–**	**↑**		**–**	**↑**	**↑**		**↑**	**↑**	**↑**		**↑**	**↑**	**↓**		↑	↓	↑		↑	↓	↑
Meg		↑	–	↑		**↑**	**↓**	**↑**		**↑**	**↑**	**↓**		↑	↓	↑		↓	↑	↑		↑	–	↓
Jade		**↓**	**↑**	**↑**		↑	↑	↓		**↑**	**↓**	**↑**		**↓**	**↑**	**↑**		**–**	**↓**	**↓**		↓	↓	↑
Kate		↑	↓	↑		**↑**	**↑**	**↑**		↑	↑	↓		↑	↓	↑		↓	↓	↑		↓	↑	↓

*Expected to increase from time 1.

**Expected to decrease from time 1.

↑ = increased from previous time.

↓ = decreased from previous time.

- = no change from previous time.

Shaded boxes indicate significant differences from previous time.

**Table 8:** Participant Development in Game Knowledge, Decision-Making, Pressure, Communication, and Psychological Constructs.

Participant	GK (SR)*				GK (MR)*				DM (SR)*				DM (MR)*				Pressure (SR)*				Pressure (MR)*				Comm (SR)*				Comm (MR)*				LoC**				OE*				PP*			
	1	2	3	4	1	2	3	4	1	2	3	4	1	2	3	4	1	2	3	4	1	2	3	4	1	2	3	4	1	2	3	4	1	2	3	4	1	2	3	4	1	2	3	4
Jamie		–	–	↑		↑	–	–		↑	–	**↓**		↑	↓	–		**–**	**–**	**↓**		↑	–	↓		↑	↓	–		**↑**		**↑**		↑	↓	↓		↑	↑	↓		↓	↑	↓
Alex		↓	↑	–		**↑**	**–**	**↑**		**↓**	**↑**	**–**		**↑**	**↑**	**–**		**↑**	**↑**	**↑**		↑	↑	–		**↑**	**↑**	**–**		**↑**	**↑**	**–**		↓	↓	–		↑	↑	–		**–**	**↑**	**↑**
Meg		–	–	–		↑	–	–		**↑**	**↓**	**↑**		**↓**	**↑**	**↓**		↑	–	–		↓	↑	–		**↑**	**↓**	**↑**		**↑**	**↑**	**–**		↓	↑	–		–	–	↑		↑	–	↑
Jade		**↑**	**↑**	**↓**		**↑**	**–**	**↑**		↑	↑	↓		**↑**	**↑**	**↑**		↓	–	↓		↑	↓	↑		**↑**	**↑**	**–**		↑	–	–		↑	–	–		**↓**	**↑**	**–**		**↓**	**↑**	**↑**
Kate		–	↑	–		–	↑	↑		**–**	**↑**	**↑**		**–**	**↓**	**↑**		**↑**	**–**	**–**		–	↑	↓		↑	↓	–		↑	↑	↓		**↑**	**↑**	**↓**		–	↓	↑		↑	↓	–

*Expected to increase from time 1.

**Expected to decrease from time 1.

↑ = increased from previous time.

↓ = decreased from previous time.

- = no change from previous session.

Shaded boxes indicate significant differences from previous time.

### Data analysis – qualitative data

To enrich the quantitative data and provide social validation of results, a strategy used in existing interventions with sports officials [e.g., 3, 54], a thematic analysis was conducted. Thematic analysis was chosen as the analytical method because of its flexibility [55] and transparent method using the six-stage process identified by Nowell et al. [56]. An independent researcher who, until this point, was unfamiliar with the HPOP programme was recruited to conduct the analysis using an inductive approach. The procedure for the analysis was thus: familiarisation with the transcripts by reviewing twice and allocating meaning units to raw data using an Excel spreadsheet (stage one). Meaning units were then used to generate initial codes (stage two). Once codes were generated, themes were identified (stage three) before another member of the research team was asked to agree or dispute any theme or code identified (stage four). Lower and higher order themes were then defined (stage five) before a meeting was held with the research team to discuss themes and their interaction between one another, as well as producing a visual representation of the themes identified (stage six).

## Results

To aid clarity in the visual analysis of each participant’s data, and to adhere to the idiographic nature of the study, the results are ordered by participant, rather than by variable [e.g., 57].

Official 1 (Jamie). From baseline to Time 4 (see Tables 2–6), Jamie showed a significant 37.5% improvement in resilience (RCI = 3.99) over the study period. Threat appraisal decreased significantly by 57.89% (RCI = −3.33). Self-rated pressure decreased by 20% (RCI = −2.27), while mentor-rated communication showed significant improvements of 25% (RCI = 2.19).

Official 2 (Alex). From baseline to Time 4 (see Tables 2–6), Alex’s resilience significantly increased by 37.5% (RCI = 3.99), with self-efficacy rising by 20.45% (RCI = 4.00), and mentor-rated self-efficacy increasing by 67.57% (RCI = 7.20). Challenge appraisal also showed a reliable increase of 22.58% (RCI = 2.38). Mentor rated game knowledge rose by 50% (RCI = 4.15). Both self-rated and mentor-rated decision-making improved reliably by 20% (RCI = 2.75) and 85.71% (RCI = 6.15) respectively. Pressure scores increased significantly, with self-rated pressure up by 33.33% (RCI = 2.27) and mentor-rated by 75% (RCI = 3.95). Communication scores improved reliably for both self (23.08% RCI = 3.16) and mentor ratings (66.67% RCI = 5.85). Functional assertiveness increased reliably by 35.48% (RCI = 3.45), along with pragmatic politeness which showed a reliable increase of 27.78% (RCI = 3.16).

Official 3: Meg. From baseline to Time 4 (see Tables 2–6), Meg’s self-rated and mentor-rated self-efficacy showed reliable increases of 16.98% (RCI = 4.00) and 26% (RCI = 3.74), respectively. Both self-rated and mentor-rated decision-making improved, with increases of 20% (RCI = 2.75) and 85.71% (RCI = 6.15) respectively. Mentor-rated pressure increased by 25% (RCI = 1.97), and communication scores increased reliably by 50% (self-rated RCI = 3.16) and 58.33% (mentor-rated RCI = 5.85).

Official 4: Jade. From baseline to Time 4 (see Tables 2–6), Jade’s resilience reliably increased by 34.62% (RCI = 3.11). Her mentor-rated self-efficacy reliably increased by 19.15% (RCI = 3.74). Challenge appraisal rose reliably by 32.26% (RCI = 3.40), and threat appraisal reliably decreased by 50% (RCI = −2.27). Both self-rated and mentor-rated game knowledge reliably increased by 18.18% (RCI = 3.46) and 25% (RCI = 2.49) respectively. Mentor-rated decision-making and mentor-rated pressure reliably improved by 30% (RCI = 3.08) and 33.33% (RCI = 1.97) respectively. Self-rated communication was reliable (RCI = 2.11) and increased by 11.11%. Finally, objective effectiveness decreased by 17.39% (RCI = −1.99), and pragmatic politeness increased by 26.09% (RCI = 3.79).

Official 5: Kate. From baseline to Time 4 (see Tables 2–6), Kate’s self-rated self-efficacy reliably increased by 14% (RCI = 3.11). Self-rated decision-making reliably increased by 18.18% (RCI = 2.75), while mentor-rated decision-making reliably decreased by 14.29% (RCI = −2.05). Self-rated pressure increased by 27.27% (RCI = 2.27), while locus of control reliably increased by 50% (RCI = 2.60).

### Qualitative results

**Fig 1:**
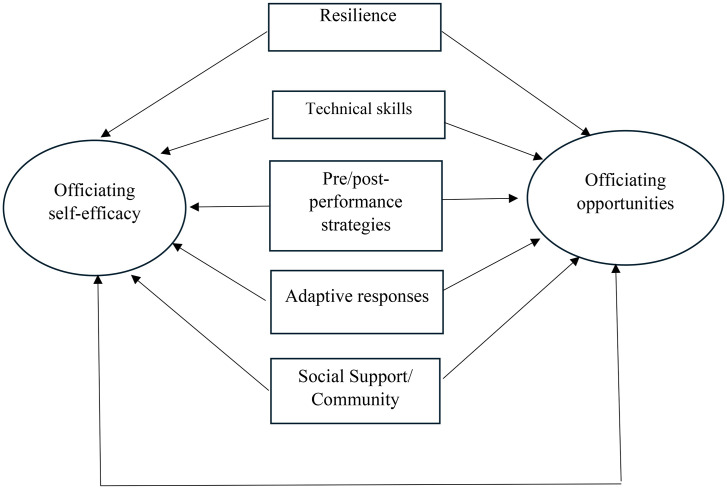
Observed higher and lower order factor structure of the HPOP.

Officials and mentors were asked whether and to what extent the official’s resilience and self-efficacy had changed since commencing the HPOP. Representative qualitative excepts from the officials and mentors were mapped to the derived lower-order factors to illustrate how participants lived experiences informed the HPOP structure (refer to Tables 9 and 10). Fig 1 shows the two higher and five lower order factors that were identified via a thematic analysis. The higher order themes were officiating self-efficacy (e.g., perceived competency and positive evaluation of officiating resources) and officiating opportunities (e.g., being offered and putting oneself forward for officiating appointments). These were reciprocal with increased officiating self-efficacy likely to increase officials wanting to take on more opportunities to officiate and undertaking more officiating opportunities is like to increase their self-efficacy. This is highlighted by Alex who stated, “I feel more confident in my abilities. I have been given opportunities to ref high level games with high level referees. Benchmarking against other referees has been good for me”. Five lower order factors were identified: re**s**ilience (e.g., recovering from negative experiences), technical skills (e.g., personal and game management), pre/post-performance strategies (e.g., development of approaches to best prepare and evaluate performance), and adaptive responses and social support/community (e.g., being proactive in their own development and recognising the importance of support). Resilience was highlighted by the quote from Jade who stated, “My resilience has grown beyond where I started. And I thought I would ever get to. I can deal with confrontation better and bounce back from any setbacks much quicker**”**. In regard to technical skills, Alex’s mentor said, “[Alex] has learned the skills to perform in a competition environment where you are backing up multiple games in a day”. Meg stated “I have specific strategies for going into my roles and am therefore better mentally prepared” which links to pre/post-performance strategies. An adaptive response is highlighted by Alex’s mentor who stated, “He [Alex] is wanting to challenge himself in high pressure situations and is seeking feedback”. Finally, social support/community is supported by the comment from Jamie who stated, “Knowing I have this kind of support around me makes me feel more confident”.

**Table 9:** Representative official quotations mapped to lower-order HPOP factors.

Lower-order factor	Representative qualitative quotes
Resilience	My willingness to put my hand up to umpire multiple top-grade games every weekend (shows increased resilience) – Jamie I am able to take on feedback and criticism without letting it have a negative affect on me – Jade I know that I now have the resilience to cope with any officiating role up to a national level – Meg
Technical skills	I have been able to identify and therefore act on areas that I can or need to improve on – Meg I believe I have the skills to achieve my goals – Jade
Pre/post-performance strategies	It has all helped me communicate and learn to move on from situations I would usually dwell on – Jamie I am using strategies to help me be more confident (e.g., focus on positives rather than dwelling on negatives) – Alex I am more conscious of mental and physical resilience [implementation of many strategies, e.g., rehearsing difficult situations] – Alex
Adaptive responses	[I] am open to new challenges and looking for opportunities to improve – Alex I believe I can take on anything that comes my way – Jade
Social support/community	Knowing I have this kind of support around me makes me feel more confident – Jamie I have also gained from all the mentors and other officials in the program – Jamie Having a structured programme with other officials really made a difference – Kate

**Table 10:** Representative mentor quotations mapped to lower-order HPOP factors.

Lower-order factor	Representative qualitative quotes
Resilience	Kate has maintained strong resilience. Meg has improved immensely…because she has taken herself out of her comfort zone and undertaken roles of which she wouldn’t ordinarily volunteer for.
Technical skills	[Alex] has learned the skills to perform in a competition environment where you are backing up multiple games in a day. Her [Jamie] growth in making big decisions is improving.
Pre/post-performance strategies	Her [Jade’s] positive self-talk and pre-game has helped [improve her self-efficacy]. Alex was hesitant as he felt he would not cope with the work load. He has learned what he needs to do to prepare.
Adaptive responses	Recently she [Jamie] did not have a good game, but sought feedback from the teams and worked with the umpire coaches to improve performance. Alex is requesting to referee harder games and believes he has the skills to adjudicate these now. Her [Kate’s] initial reaction [to a negative event] was to go straight home…within five minutes she was discussing what happened and ‘next time’…
Social support/community	She [Kate] now articulates a sense of belonging in the team of referees.

## Discussion

The aim of the present study was to examine the effects of the HPOP program on the self-efficacy, resilience, challenge and threat appraisals, physical and mental preparation, locus of control, and functional assertiveness (including objective effectiveness and pragmatic politeness) of participating officials in the 2021 cohort. Results indicate positive change for the most part, with data analyses highlighting meaningful increases in self-efficacy, resilience, physical and mental preparation, challenge appraisal, and pragmatic politeness. However, significant change did not occur for threat appraisal, locus of control, and objective effectiveness.

The significant increase in resilience from pre- to post-program was an expected result, because part of the program was aimed at providing protective factors for psychological resilience, including social support, the environment, and metacognitive appraisal [58]. An important aspect to consider, is the potential similarity between resilience and challenge appraisal which concern the relevance that individuals attribute to stressful situations and the potential consequences for their well-being [10]. The link between resilience and challenge appraisal has been highlighted in a number of studies [e.g., 59, 60]. For example, individuals with high resilience may be more likely to appraise stressful situations as a challenge rather than a threat, likewise, seeing stress as an opportunity rather than a problem may contribute to greater resilience over time. In the current study, while challenge appraisal increased for all officials, threat appraisal was unchanged for three officials, but decreased for Jamie and Jade, possibly because they were no longer officiating, due to the off-season and did not appraise any upcoming threat. In contrast, while Alex and Kate’s threat appraisal had decreased during the program, it had subsequently increased (not at a significant level) at the final testing phase. Both officials were still officiating and had interstate competitions coming up that were at a higher level than what they were currently doing, potentially increasing threat appraisal. The fact that challenge and threat did not change in tandem in opposing directions perhaps indicates that these appraisal constructs are not dichotomous [61], and should be measured separately as is done in much recent research [e.g., 23].

Like resilience and challenge appraisal, there was an increase in self-efficacy across the program, which could be attributed to a range of factors as self-efficacy is “influenced by mastery experiences, referee knowledge/education, support from others, physical/mental preparedness, environment comfort, and perceived anxiety” [26, p.1], all of which were covered across the HPOP. For example, an important source of self-efficacy is seeing other people succeed, which raises their belief that they also have the capabilities to master comparable activities when they are given similar opportunities [62]. While four of the official’s demonstrated increased self-efficacy across the program, the one official whose self-efficacy did not increase (Jamie) commenced the program with a relatively high self-efficacy score. Notably, Jamie’s initial score for self-rated self-efficacy was considerably higher from their mentor’s rating at the commencement of the program, suggesting a possible overestimation of self-efficacy at the initial testing point. Conversely, Jamie’s qualitative data highlights positive perceived changes in self-efficacy over time. Specifically, at time 2, Jamie stated: “…my self-efficacy has definitely grown. Knowing that I have this kind of support (mentor and access to other support) around me makes me feel more confident and makes me believe in myself more”.

Looking at the subscales of the REFS, apart from Meg, all officials developed their game knowledge across the program. This is not unexpected as all the official’s spent time developing this through discussions with their mentors, other officials, and also in workshops. In comparison, decision making outcomes were more variable. Although most officials improved, both Jamie and their mentor perceived no significant overall change in Jamie’s decision-making. This perception aligns with their quantitative data, which showed increases at times 2 and 3 followed by a decline at time 4. This may have been as a result of their sporting season moving to the off-season approximately two months before the final testing and that they had ceased officiating. Additionally, this may reflect the results for the pressure subscale for both Jamie and Jade, as neither were officiating at the time 4. With the final REFS subscale of communication, all officials improved over the four-time frames. This may have been due to a general increase in communication and an awareness of the importance of communicating.

For physical and mental preparation (PMP), three officials demonstrated improvement across the program. In contrast, Jamie and Kate showed no overall change from the start to the completion of the program, although both, along with Meg peaked at either time 2 and/or 3. Physical and mental preparation had been identified as an important factor for officials [e.g., 2]. The observed improvements may have occurred for a number of reasons. Firstly, although fitness levels were not directly measured, participants may have increased their knowledge related to fitness and preparation. Secondly, fitness-related goals were established at the commencement of the program which may have supported development in this area. For the two participants whose PMP scores overall did not change, different explanations are evident. Jamie commenced the program with a relatively high level of PMP, thereby, possibly limiting further improvement. In contrast, Kate officiates in a sport where high levels of fitness are not required, which may have resulted in less focus in this area which subsequently influenced her PMP outcomes.

For objective effectiveness, Jade was the only official who reported a significant decrease, and for pragmatic politeness, there was a significant increase across the program for Jamie, Alex, Meg and Jade. There are several potential reasons why pragmatic politeness may have improved. Firstly, as officials gained more experience throughout the program, they likely became more comfortable in their roles, which could have contributed to greater confidence in their interpersonal communication and assertiveness. Secondly, if the mentors played an active role in guiding officials, their feedback and support could have contributed to improvements in social communication, particularly in terms of assertiveness and politeness. Further, officials likely observed and interacted with their peers throughout, and seeing how others handled assertiveness in officiating situations may have helped individuals adapt their own communication strategies. The program itself may have facilitated informal (and formal) learning opportunities that contributed to these improvements.

Having considered the quantitative data, the qualitative data revealed that there were two higher order themes of officiating opportunities and officiating self-efficacy which were linked. It is unclear where officiating opportunities impacts self-efficacy or vice versa, it is more likely that there is a reciprocal interaction between these factors. Officials described how increased opportunities supported confidence development, while greater self-efficacy, in turn, shaped their willingness to engage more fully in further officiating opportunities. This is not surprising, given that, mastery experiences, namely doing the task, according to Bandura [62] represents the strongest source of self-efficacy. Accordingly, higher self-efficacy may both result from, and facilitate access to, increased officiating opportunities. Supporting this interpretation, Grylls et al. [2] demonstrated that officiating self-efficacy was predicted by the number of games officiated, reinforcing the role of opportunity as a key confidence building mechanism.

Further examination of this interaction identified five lower-order themes: resilience, technical skills, pre- and post-performance strategies, adaptive responses, and social support/community, which appeared to underpin both higher-order themes. Collectively, these lower-order themes function as mechanism through which officiating opportunities and officiating self-efficacy are mutually reinforced within the HPOP framework. Importantly, the findings suggest that officiating opportunities in isolation may be insufficient to enhance self-efficacy unless they are accompanied by appropriate psychological and social supports.

Specifically, resilience has been shown to support self-efficacy and enables a stable sense of capability [2]. This is reinforced by Fletcher and Sarker [63] who conceptualised resilience as enabling performers to maintain confidence and functioning across adversity. Further, resilient officials are more likely to persist in officiating over time and accumulate experience and developmental opportunities [2,64]. In regard to technical skills, which include game knowledge, communication and decision making, can directly inform officials belief that they can execute the role effectively [26,46], with mastery experiences strengthening confidence, particularly under pressure [62]. Officials perceived as technically competent are also more likely to be allocated higher level matches with increased officiating opportunities [2]. Pre- and post-performance strategies further enhance preparedness, perceived control, and learning reinforcing confidence even after errors [2,62,65]. Adaptive responses, such as resetting after mistakes and maintaining task focus, protect self-efficacy [29], allowing officials to learn they can manage adversity not just in only ideal conditions [66]. Finally, social support reinforces self-efficacy through encouragement, feedback and modelling, while reducing the perceived risk associated with engaging in challenging officiating opportunities [62,67]. Further, mentoring and perceived support are crucial for retaining officials in challenging environments [64], thereby potentially leading to officiating opportunities.

One question this paper has raised is around the mentor/mentee relationship, specifically the differences in the ratings between the official and mentor and how in fact the differences lessened over the times. It could be suggested that the mentors may have initially provided harsher evaluations because they are still getting to know their mentees and have not yet established a strong working relationship. As the year progresses and they spend more time together, trust and understanding develop, leading to more aligned assessment. This suggests that the program could benefit from fostering a stronger mentor/mentee connection earlier in the process, which might enhance engagement and accelerate development. This is reinforced by Hancock and colleagues [68] who examined ice-hockey officials’ perceptions of group processes and advocated for the establishment of mentorship structures, whereby experienced officials are paired with less-experienced newcomers.

Several limitations should be highlighted in the current study. Firstly, the sample size is small and potentially unbalanced by gender, and due to the nature of the of the study, being inferential, we cannot generalise the results to other officials. Secondly, it is hard to link specific causation in the development of the measured factors. For example, improved decision making may be linked to increased physical fitness rather than specific decision-making skills, as suggested by several studies [e.g., 69, 70], which indicate a direct link between the level of physical conditioning and decision making. Likewise, Castillo-Rodríquez et al. [71] state in their study on soccer referees, that performance on physical tests could be a good predictor of success in other behaviours of the selected officials. Further, several interview questions were framed around demonstrating changes in self-efficacy and resilience. While this reflected the study’s focus on these targeted outcomes and assisted in maintaining a structured interview format, it may have encouraged participants and mentors to emphasise positive changes and limited discussion of unchanged or negative experiences. Additionally, the involvement of the lead author in delivering several HPOP sessions may introduce biases. We recommend that future research employ more neutral interview questions to allow for a broader range of participant responses, and has an independent interview undertake the data collection. Lastly, it is difficult to attribute changes in variables directly to specific HPOP content, as the program is multimodal and multidisciplinary.

Whilst there were several limitations, there were also a number of notable strengths associated with this study. Firstly, it is first study of its kind to examine the psychosocial impact on the HPOP program on sports officials, contributing novel insights to the existing literature. The study also has benefits from a rich dataset, incorporating both quantitative and qualitative (idiographic) data, which allows for a more nuanced understanding of individual experiences and changes over time. Further, the inclusion of a wide range of psychological variables provides a comprehensive assessment of the program’s effects, capturing multiple facets of officials’ development beyond just performance-related outcomes. Additionally, the results offer preliminary evidence that the HPOP program can positively influence key psychological factors, such as resilience and self-efficacy, highlighting its potential as an effective intervention. Finally, the use of mixed-methods approach strengthens the study by integrating statistical findings with detailed qualitative insights, enabling a deeper exploration of the mechanisms underlying observed changes. This methodological rigour ensures that the results are not only statistically meaningful but also contextually rich, providing valuable implications for both researchers and practitioners working with sports officials.

In conclusion, the current research contributes to the growing body of literature on the psychological development of sports officials, offering valuable insights into the role of structured support programs such as HPOP. By examining key psychological variables – including resilience, self-efficacy, cognitive appraisal, and decision-making – the study highlights how targeted interventions can foster mental preparedness and overall officiating performance. The findings underscore the importance of providing officials with psychological tools and social support to navigate the demands of their roles effectively. Future studies and applied workshops for officials may consider incorporating behavioural strategies aligned with the HPOP. These could include designing structured opportunities for officials to work and develop together within officiating programs, with the aim of enhancing self-efficacy sand increasing willingness to engage in officiating roles. Workshops may also focus on developing pre- and post-performance routines, tactical skills, and resilience related competencies. Importantly, programs should provide officials with opportunities to operate outside their comfort zones within the safety of a structured mentoring framework. Further, explicitly teaching officials to appraise stressful situations as challenges rather than threats may be beneficial. Additionally, the use of a mixed-methods approach ensures that both quantitative trends and individual experiences are captured, reinforcing the real-world applicability of the results. While further research with larger and more diverse samples is needed, this study lays the foundation for future investigations into officiating psychology and informs the design of programs aimed at enhancing officials’ psychological resilience, confidence, and decision-making capabilities. Ultimately, the research has meaningful implications for sports organisations, educators, and policymakers seeking to improve the training and well-being of officials across various levels of competition.

## Supporting information

S1 Dataset Data for quantitative analyses. (XLSX)
