# Supplementary material for: Evaluating the psychosocial effects of the Northern Territory Institute of Sport (NTIS) High Performance Officiating Program (HPOP)
Source: PLoS One. 2026 Aug 5;21(8):e0354971. doi: 10.1371/journal.pone.0354971 (PMC13440864; doi:10.1371/journal.pone.0354971)
Supplement: Supplementary file 2 [file pone.0354971.s004.docx]

**Responses to Reviews**

**Manuscript No. PONE-D-25-62568**

**Evaluating the psychosocial effects of the Northern Territory Institute of Sport (NTIS) High Performance Officiating Program (HPOP)**

**PLOS ONE**

Firstly, let us thank the Co-Editors and the reviewers for taking the time to review the manuscript and for providing thoughtful and constructive comments. We have endeavoured to address each comment and have shown the changes to the manuscript in red text.

**Reviewer #2 comments:**

Abstract: Please review the abstract to ensure it aligns with the results that are presented. There is language around “…increases/improvements, but only XX participants had reliable increases.” This is confusing and does not align with the results, which the authors noted now only focus on significant/reliable changes.

Thank you for this suggestion. We agree that the previous wording could be interpreted as implying improvements across all participants. The abstract has been revised to focus on reliable improvements identified through the Reliable Change Indices (RCIs), with self-efficacy and resilience findings now reported in a manner consistent with the results section. Additional wording was included to acknowledge the variability of responses across individual officials.

2. P2, line 23: Suggest changing “However” to “Further”, since emphasizing that only one program existed for a twelve-month period at the time aligns with the previous sentence about there being few (limited) training programs for officials. Currently it reads like a contrast, but that seems incorrect.

Page 2, line 23. Changed “However” to “Further”.

Also suggest inserting the year(s) when this program occurred here, since it’s not clear to the reader at this point in the paper when this 12-month period occurred.

Page 2, line 23. We have added the year (2021) at the first mention of the NTIS High Performance Officiating Program (HPOP).

3. P6, line 6: I think “psychological” resilience here.

Page 6, line 6. We have changed “Psychology” to “Psychological”.

4. P6, line 11: Suggest comma after TCTSA.

Page 6, line 11, have added a comma after TCTSA.

5. P6, lines 14-18: Revise, some issues with the sentence structure. 

Page 6, lines 15-18. Thank you for highlighting this issue. The sentence has been revised to improve clarity, grammar, and flow to articulate the relevance of challenge-threat theory to non-athlete performance settings, including sports officials.

6. P10, qualitative questions: Why do the questions for both officials and mentors imply that official self-efficacy and resilience must have increased? (e.g., Please give an example of how you have shown increased self-efficacy since commencing in the HPOP?). This choice of wording would certainly have the potential to bias the results.

Thank you for raising this important point. We acknowledge that the wording of some interview questions may have encouraged participants and mentors to reflect specifically on positive changes in self-efficacy and resilience. The interviews were intentionally focused on exploring these constructs, as they were key target outcomes of the HPOP, however, we recognise that the phrasing may have introduced a degree of positive-response bias and may have limited opportunities for participants to discuss no change or negative experiences. This limitation has now been acknowledged in the discussion section (Page 23, lines 17-24), and we recommend that future research employ more neutral interview questions to allow for a broader range of participant responses.

7. P13-14, qualitative data analysis: The six stages of data analysis are poorly written. Please revise.

We have revised this section as requested and, while the original writing represented how the stages were presented by Nowell et al. (2017), we have written the stages in a more continuous prose to promote fluency. (Page 15, lines 2-10).

8. P14-15, quantitative results: I’m a bit confused with some of the subscales/factors that show up in the results and the tables (e.g., functional assertiveness subscales) but don’t appear to be clearly highlighted as distinct subscales earlier in the measures section. Please ensure all subscales that are mentioned in the results and tables are clearly identified within the measures section (so that the reader knows in advance exactly what subscales to expect later in the results).

Page 13, lines 3-7. Thank you for this observation. We have revised the method section to clearly identify and describe the two subscales as reported in the results and tables.

9. P15-16, qualitative results: I realize there is a desire to keep the qualitative section shorter, but I find the results rather thin on detail and context. In particular, the lower-order themes are not actually described. Rather, they are merely stated with a representative quote. At minimum, there needs to be a brief description of what these themes mean (ie., define the themes).

Thank you for this recommendation that, we believe, has enhanced the detail of the qualitative results. Amendments have been made on page 17 (lines 5-11 and lines 13-18) where definitions of the lower order and higher order themes are defined.

10. P16, lines 7-9: Incomplete sentence regarding lower-order themes.

Thank you for highlighting this issue. The incomplete sentence as been corrected and revised to clearly describe the lower-order themes identified within the qualitative analysis.

11. P20, line 12: Period in middle of sentence.

Fixed as requested
