# Supplementary material for: Evaluating the psychosocial effects of the Northern Territory Institute of Sport (NTIS) High Performance Officiating Program (HPOP)
Source: PLoS One. 2026 Aug 5;21(8):e0354971. doi: 10.1371/journal.pone.0354971 (PMC13440864; doi:10.1371/journal.pone.0354971)
Supplement: Supplementary file 3 [file pone.0354971.s003.docx]

**Responses to Reviews**

**Manuscript No. PONE-D-25-62568**

**Evaluating the psychosocial effects of the Northern Territory Institute of Sport (NTIS) High Performance Officiating Program (HPOP)**

**PLOS ONE**

Firstly, let us thank the Co-Editors and the reviewers for taking the time to review the manuscript and for providing thoughtful and constructive comments. We have endeavoured to address each comment and have shown the changes to the manuscript in red text.

**Reviewer #1 comments:**

The manuscript reads very well and highlights the results of a training program for sporting officials, an underdeveloped area in sport psychology literature. Authors managed to provide information on the quantitative and qualitative evaluation of the program, emphasising certain components.

We thank the reviewer #1 for taking the time to provide very detailed comments on our manuscript and we have endeavoured to address all of their points.

**Specific comments**

Literature Review

It is informative and provides an adequate level of detail around the performed evaluation of the intervention program.

However, since the HPOP intervention contents haven't been published yet, authors need to provide more details of the actual intervention, either within the text or on Table 1. Authors need to define better the contents of workshops (i.e. conflict resolution, self-efficacy) and, the contents of the discussions. It would also be useful to clarify which components of the program were evaluated as more helpful by the participants and their trainers/mentors.

In response, we have substantially expanded the description of the HPOP. Table 1 (page 34) now provides a detailed overview of session content, including workshop topics (e.g., resilience, self‑efficacy, conflict resolution, pressure management) and the focus of facilitated discussions. Additionally, clarification has also been added within the text to highlight program components that were perceived as particularly valuable based on participant and mentor feedback. For example, the increased opportunity to officiate (page 16, lines 7-9), and physical and mental preparation (Page 19, lines 8-10),

Results

In tables 2-6 it would be helpful to add vertical lines among measures and also highlight (i.e. bold fonts) the significant results among measures. These would help the reader appreciate better each measurement, changes occurring and where the significant results can be found.

We have revised Tables 2–6 accordingly. Vertical lines have been added to clearly separate constructs, and statistically reliable changes (based on the Reliable Change Index criterion) are now highlighted using bold formatting to aid readability.

Qualitative results p.16 (line 5): Authors tried to present their qualitative results within the manuscript. However, they are quite lengthy and hinder the reader's understanding of the type and details of these findings.
Authors need to run a thematic analysis based on the results of the qualitative data and present themes, subthemes and quotes accordingly. Adding a table or two would also help the reader understand better the nature of the qualitative findings.

Thank you for the suggestion. We thought about the discussed the reviewer’s comment a lot, and decided to bring onto the project a colleague to undertake a more substantial and meaningful analysis of the qualitative data. This allowed the data to be view more objectively and has added more value to the paper. Thus, we have conducted a thematic analysis and presented both higher and lower themes through a Figure. Appropriate quotes have been used, and we have also added two tables with representative quotes of the lower order themes, whilst being careful not to add too much more text to the paper.

Discussion

Since the evaluation relates to the High Performance Officiating Program (HPOP) it is important to include a section on which parts of the program, its delivery and its supporting agents were found mostly effective vs. not useful, by participants and stakeholders. This means that authors need to look at the trends in the quantitative data or examine the qualitative data with this glance. Such emphasis will highlight the potential and the strongest areas of the program, creating an important reference point and comparator for the future.

Based on the reviewers comments we have discussed the strengths of the program through the thematic analysis. For example, Figure 1 (page 44), shows that social support was an important lower order factor, likewise, quotes such as “I have also gained from all the mentors and other officials in the program” (page 42, table 9). All of the lower-order factors have been identified in the paper, through the results, discussion, tables (9-10) and Figure 1.

**Reviewer #2 Comments**

This study explored the psychosocial effects of a high-performance officiating program on five officials in Australia. There are useful insights that would be relevant to several stakeholders interested in improving the experiences of officials. Use of both quantitative and qualitative analyses yield some robust findings. Nonetheless, some weaknesses include: lack of clarity around the theoretical underpinnings of the constructs examined (why they were selected), lack of clarity around who oversees the training program for officials, commenting on unreliable (nonsignificant) quantitative results, and lack of information regarding the qualitative methods used and presentation of qualitative results. The quality of writing is generally adequate, though sentence structure is poor in some areas of the Discussion in particular. In my view this research has the potential to practically impact officiating training programs and the experiences of officials.

We thank the reviewer #2 for taking the time to provide very detailed comments on our manuscript and we have endeavoured to address all of their points to improve theoretical grounding, methodological clarity and overall readability of the manuscript.

**Specific comments**

Introduction:
1. P3, lines 12-13: This quote about coaches being considered performers, just like their athletes, seems out of place. The sentences before it and after it emphasize the extent of research on officials, therefore a sentence (quote) strictly about coaches and athletes sandwiched in-between detracts from the flow of writing and focus on sport officials.

P2, lines 13-15. Thank you for the feedback. The quoted sentence has been removed, and the surrounding text has been revised to maintain focus on sport officials. Reference numbering has been updated accordingly.

2. P3-4, starting line 24: Was or still is the only program for sport officials in Australia? Some clarification would be helpful as using the past tense to refer to the program but then describing the program in the present tense is a bit confusing.

P2-3, starting line 23. The relevant section has been revised to clarify that the HPOP represents the only high-performance officiating program catering for a range of sporting officials at the time. Tense usage has been corrected for consistency (page 3, lines 2-4).

3. P4, lines 10-13: Can you situate the goals of this study within the some of the relevant theories/models or previous literature on coach officiating as outlined in a recent review by Hancock and Pizzera (2025)? Saying that you will examine resilience, challenge and threat evaluation, and self-efficacy because you previously examined those psychological markers in tennis is fine, but it lacks some connection to the broader areas examined in the literature on sport officials. For instance, Hancock and Pizzera outline relevant models/ theories but also key “research areas” such as officials’ mental skills, motivations, and decision-making.

Hancock & Pizzera (2025). The psychology of sport officiating. Psychology of Sport and Exercise.

Page 6, Lines 1-18. Thank you for providing the reference. We have strengthened the theoretical framework by explicitly positioning the study within the Theory of Challenge and Threat States in Athletes (TCTSA). This now clearly explains how the selected constructs align with key theoretical and applied research.

4. P5, lines 3-19: A brief yet clear explanation of what challenge and threat states/appraisals actually mean seems to be absent from this paragraph.

Page 4, lines 4-7. A concise explanation of challenge and threat appraisals has been added to the introduction to ensure conceptual clarity for readers.

5. P5, line 24-25: To clarify, there are no known previous studies exploring sport officials’ experiences in training programs–in Australia or elsewhere? Or is it that these specific psychological constructs have not been examined among officials in training programs? If there has been any previous research examining officials’ experiences in training programs, it would be useful for readers to know this and for the authors to highlight this.

Page 6, lines 19-20. We now explicitly state that this study represents the first empirical evaluation of the HPOP, addressing this point directly.

6. P6-7, starting line 22: The authors are investigating many factors: self-efficacy, resilience, challenge and threat appraisals, physical and mental preparation, locus of control, and functional assertiveness. Similar to my comment earlier (#2) about linking the study’s aims within a particular theory/model or key “research area/topic”, it would be helpful to articulate why these various factors are being investigated together and how they fit together from a theoretical and/or research standpoint when presenting the overarching purpose of the study. Some more emphasis on the theoretical underpinnings of this study would strengthen the manuscript.

Page 6, lines 1-18. Thank you for the feedback. We have now clearly articulated how the study’s constructs collectively aligns with the TCTSA framework, including previous research.

Methods:

7. P7, line 5: Why were 6 officials initially selected? What is the justification for selecting this number of participants? It’s a small number of participants for a study, but I appreciate the applied nature of this work. Regardless, why did the program consist of 6 – and ultimately 5 – individuals?

Page 7, lines 1-2. All officials who applied for the HPOP were included in the study. An explanation has been provided regarding the selection of the officials.

8. P7, HPOP schedule: Is the high-performance officiating program governed/managed by a university (and this study’s authors) or a government-related sport agency? I understand earlier in the Introduction there is some mention of the program being offered by the Northern Territory Institute of Sport – but it’s not clear whether this is through a university or government sporting body. Some clarification here or in the Introduction is likely warranted and would also help readers understand why a relatively small number of officials were included in this program and why particular themes were targeted (e.g., resilience). Some additional context would be helpful to understand to what extent the authors developed this program or contributed to it.

Pages 7-8, starts on line 22. The methods section now clarifies that the HPOP was designed and delivered by the Northern Territory Institute of Sport (NTIS), with the research team evaluating the program rather than governing it.

9. P10, line 7: What were the qualitative questions for officials at time 1?

Page 10, lines 7-16. No qualitative questions were asked at Time 1. This has been fixed. All qualitative questions asked of the officials and mentors at later time points have been explicitly listed and redundant statements have been removed.

10. P10, lines 15-16: What were the qualitative questions asked to mentors about their perceptions of their officials’ resilience and self-efficacy at T2, T3, an T4? Can also remove statement about questions being the same as those from previous timepoint since qualitative questions were evidently not asked to mentors at T1.

Page 10, lines 17-23. The qualitative questions have been added, likewise the statement has been removed.

11. P13, lines 9-10: How were the qualitative data analyzed and coded? What was the method used? I realize this section will not be as large as we would expect to see for a dedicated qualitative study, but there is no information about how the qualitative data were analyzed (e.g., inductive vs. deductive) and if (even briefly) any criteria were adopted to ensure quality standards.

Page 13-14. Starts on line 17. Thank you for the feedback, we have undertaken a thematic analysis (as per reviewer 1’s comments) with detailed information on how it was undertaken in this section.

Quantitative results:

12. I’m wondering whether it’s appropriate to comment on construct increases/decreases that were deemed not reliable based on the RCI. Since several constructs across the five participants did increase/decrease reliably, commenting on the unreliable increases/decreases seems questionable. Similar to traditional non-significant p-values where we would typically not make claims about increases/decreases (or relationships) that are non-significant, why comment on unreliable findings here as being meaningful? Since there are several reliable findings to draw on anyways, my suggestion would be to rely only on those (which puts more confidence in the findings and enhances the quality of the research and associated claims).

Pages14-15, starts on line 7. All non-reliable changes have now been removed. The results and discussion sections focus exclusively on reliable RCI findings to strengthen the validity of claims.

Qualitative results:

13. P16, resilience: Jade’s quotes regarding perceptions of resilience are repeated verbatim at different time points – this seems unlikely.

Due to the changes made in regard to the qualitative analysis, Jade’s comments have been removed as they no longer fit within the article.

14. P18, line 13: This paragraph seems like you are introducing the qualitative findings on self-efficacy for the first time here. However, this is the third paragraph about this finding that was introduced on P17, and it is repetitive. Overall, I would encourage the authors to review their qualitative findings and look for areas to enhance the flow of writing – ensuring results are succint and not repeated.

As noted previously we have reviewed the qualitative findings to enhance the flow of the writing.

Discussion:

15. P22, 11-18: Since there is not much emphasis in the paper on how the mentor and mentee interacted during the program (e.g., how often, topics discussed), this paragraph could be a good opportunity to relate the findings to previous research that has pointed to the value of mentee-mentor relationships among officials. For instance, you could point to previous research that highlighted the important role of senior officials in mentoring younger officials in ice hockey (Hancock et al., 2018).

Page 21, lines 22-25. Thank you for the reference, the discussion now explicitly links findings to prior mentoring research in officiating, including work by Hancock et al (2018), highlighting the role of mentor-mentee relationship.

Hancock, D. J., Martin, L. J., Evans, M. B., & Paradis, K. F. (2018). Exploring Perceptions of Group Processes in Ice Hockey Officiating. Journal of Applied Sport Psychology, 30(2), 222-240. [https://doi.org/10.1080/10413200.2017.1349208](https://aus01.safelinks.protection.outlook.com/?url=https%3A%2F%2Fdoi.org%2F10.1080%2F10413200.2017.1349208&data=05%7C02%7Cliz.grylls%40cdu.edu.au%7Cb32c9d5566384ca2be0908de9099b801%7C9f2487678e1a42f3836fc092ab95ff70%7C0%7C0%7C639107187635347834%7CUnknown%7CTWFpbGZsb3d8eyJFbXB0eU1hcGkiOnRydWUsIlYiOiIwLjAuMDAwMCIsIlAiOiJXaW4zMiIsIkFOIjoiTWFpbCIsIldUIjoyfQ%3D%3D%7C0%7C%7C%7C&sdata=vJ%2B0k74ur1qNl6bpsAKLCxlJeRb9AU6gqnAmTl4pjGE%3D&reserved=0)

Minor comments:
16. No need for period after title.

17. Headings do not follow PLOS One formatting guidelines.

18. P4, line 16: “Positively”?

19. P6, line 2: “Importance”?

20. P6, lines 7-9: Awkward sentence structure. Please re-phrase.

21. P9, line 23: Obtaining ethical approval was already mentioned earlier in the Methods. Pick one place to make this statement.

22. P9, line 24: Data were collected.

23. P10, line: Complete “it”? Something is missing from this sentence.

24. P16, line 6: “to” what

25. P16, lines 20-24: Abrupt transition to Alex’s mentor’s views at Time 4, then moving to Alex’s (official) views at time 2. This seems out of order and a bit hard to follow.

26. P16, line 25: “This is reinforced by Meg (at time 2) stated…” Sentence structure error here.

27. P20, lines 23-25: This is an incomplete sentence.

28. P21, lines 4-7: Long, choppy sentence. Please revise.

29. P21, lines 15-19: Long, choppy sentence. Please revise.

30. P21, 19-20: Unclear what this sentence means.

31. P22, lines 11-13: A comma is needed after “relationship.”

All minor editorial and formatting comments (e.g. punctuation, sentence structure, heading format, clarity issues, and duplication) have been addressed. Specific sentences have been revised or removed as requested, and the manuscript has been carefully edited for clarity and adherence to PLOS One formatting guidelines.
