# Supplementary material for: Evaluating the psychosocial effects of the Northern Territory Institute of Sport (NTIS) High Performance Officiating Program (HPOP)
Source: PLoS One. 2026 Aug 5;21(8):e0354971. doi: 10.1371/journal.pone.0354971 (PMC13440864; doi:10.1371/journal.pone.0354971)
Supplement: Peer review history

# Peer review: Decision Letter 0

**DOI:** 10.1371/journal.pone.0354971.r001

**Authors:** Ender Senel

31 Mar 2026

PONE-D-25-62568

Evaluating the psychosocial effects of the Northern Territory Institute of Sport (NTIS) High Performance Officiating Program (HPOP)

PLOS One

Dear Dr. Turner,

Thank you for submitting your manuscript to PLOS ONE. After careful consideration, we feel that it has merit but does not fully meet PLOS ONE’s publication criteria as it currently stands. Therefore, we invite you to submit a revised version of the manuscript that addresses the points raised during the review process.

Please submit your revised manuscript by May 15 2026 11:59PM. If you will need more time than this to complete your revisions, please reply to this message or contact the journal office at plosone@plos.org. When you're ready to submit your revision, log on to https://www.editorialmanager.com/pone/ and select the 'Submissions Needing Revision' folder to locate your manuscript file.

Please include the following items when submitting your revised manuscript:

- A letter that responds to each point raised by the academic editor and reviewer(s). You should upload this letter as a separate file labeled 'Response to Reviewers'.
- A marked-up copy of your manuscript that highlights changes made to the original version. You should upload this as a separate file labeled 'Revised Manuscript with Track Changes'.
- An unmarked version of your revised paper without tracked changes. You should upload this as a separate file labeled 'Manuscript'.

If you would like to make changes to your financial disclosure, please include your updated statement in your cover letter. Guidelines for resubmitting your figure files are available below the reviewer comments at the end of this letter.

If applicable, we recommend that you deposit your laboratory protocols in protocols.io to enhance the reproducibility of your results. Protocols.io assigns your protocol its own identifier (DOI) so that it can be cited independently in the future. For instructions see: https://journals.plos.org/plosone/s/submission-guidelines#loc-laboratory-protocols. Additionally, PLOS ONE offers an option for publishing peer-reviewed Lab Protocol articles, which describe protocols hosted on protocols.io. Read more information on sharing protocols at https://plos.org/protocols?utm_medium=editorial-email&utm_source=authorletters&utm_campaign=protocols.

As the corresponding author, your ORCID iD is verified in the submission system and will appear in the published article. PLOS supports the use of ORCID, and we encourage all coauthors to register for an ORCID iD and use it as well. Please encourage your coauthors to verify their ORCID iD within the submission system before final acceptance, as unverified ORCID iDs will not appear in the published article. *Only*  the individual author can complete the verification step; PLOS staff *cannot*  verify ORCID iDs on behalf of authors.

We look forward to receiving your revised manuscript.

Kind regards,

Ender Senel, PhD

Academic Editor

PLOS One

Journal Requirements:

When submitting your revision, we need you to address these additional requirements.

1. Please ensure that your manuscript meets PLOS ONE's style requirements, including those for file naming. The PLOS ONE style templates can be found at

https://journals.plos.org/plosone/s/file?id=wjVg/PLOSOne_formatting_sample_main_body.pdf and

https://journals.plos.org/plosone/s/file?id=ba62/PLOSOne_formatting_sample_title_authors_affiliations.pdf

2. Please include a complete copy of PLOS’ questionnaire on inclusivity in global research in your revised manuscript. Our policy for research in this area aims to improve transparency in the reporting of research performed outside of researchers’ own country or community. The policy applies to researchers who have travelled to a different country to conduct research, research with Indigenous populations or their lands, and research on cultural artefacts. The questionnaire can also be requested at the journal’s discretion for any other submissions, even if these conditions are not met.  Please find more information on the policy and a link to download a blank copy of the questionnaire here: https://journals.plos.org/plosone/s/best-practices-in-research-reporting. Please upload a completed version of your questionnaire as Supporting Information when you resubmit your manuscript.

3. Please include captions for your Supporting Information files at the end of your manuscript, and update any in-text citations to match accordingly. Please see our Supporting Information guidelines for more information: http://journals.plos.org/plosone/s/supporting-information.

4. We note that there is identifying data in the Supporting Information file Dataset.xlsx. Due to the inclusion of these potentially identifying data, we have removed this file from your file inventory. Prior to sharing human research participant data, authors should consult with an ethics committee to ensure data are shared in accordance with participant consent and all applicable local laws.

Data sharing should never compromise participant privacy. It is therefore not appropriate to publicly share personally identifiable data on human research participants. The following are examples of data that should not be shared:

-Name, initials, physical address

-Ages more specific than whole numbers

-Internet protocol (IP) address

-Specific dates (birth dates, death dates, examination dates, etc.)

-Contact information such as phone number or email address

-Location data

-ID numbers that seem specific (long numbers, include initials, titled “Hospital ID”) rather than random (small numbers in numerical order)

Data that are not directly identifying may also be inappropriate to share, as in combination they can become identifying. For example, data collected from a small group of participants, vulnerable populations, or private groups should not be shared if they involve indirect identifiers (such as sex, ethnicity, location, etc.) that may risk the identification of study participants.

Additional guidance on preparing raw data for publication can be found in our Data Policy (https://journals.plos.org/plosone/s/data-availability#loc-human-research-participant-data-and-other-sensitive-data) and in the following article: http://www.bmj.com/content/340/bmj.c181.long.

Please remove or anonymize all personal information Dataset.xlsx, ensure that the data shared are in accordance with participant consent, and re-upload a fully anonymized data set. Please note that spreadsheet columns with personal information must be removed and not hidden as all hidden columns will appear in the published file.

5.  If the reviewer comments include a recommendation to cite specific previously published works, please review and evaluate these publications to determine whether they are relevant and should be cited. There is no requirement to cite these works unless the editor has indicated otherwise.

[Note: HTML markup is below. Please do not edit.]

Reviewers' comments:

Reviewer's Responses to Questions

**Comments to the Author**

1. Is the manuscript technically sound, and do the data support the conclusions?

The manuscript must describe a technically sound piece of scientific research with data that supports the conclusions. Experiments must have been conducted rigorously, with appropriate controls, replication, and sample sizes. The conclusions must be drawn appropriately based on the data presented.

Reviewer #1: Yes

Reviewer #2: Partly

**********

2. Has the statistical analysis been performed appropriately and rigorously?

Reviewer #1: Yes

Reviewer #2: Yes

**********

3. Have the authors made all data underlying the findings in their manuscript fully available?

The PLOS Data policy requires authors to make all data underlying the findings described in their manuscript fully available without restriction, with rare exception (please refer to the Data Availability Statement in the manuscript PDF file). The data should be provided as part of the manuscript or its supporting information, or deposited to a public repository. For example, in addition to summary statistics, the data points behind means, medians and variance measures should be available. If there are restrictions on publicly sharing data—e.g. participant privacy or use of data from a third party—those must be specified.

Reviewer #1: Yes

Reviewer #2: Yes

**********

4. Is the manuscript presented in an intelligible fashion and written in standard English?

PLOS ONE does not copyedit accepted manuscripts, so the language in submitted articles must be clear, correct, and unambiguous. Any typographical or grammatical errors should be corrected at revision, so please note any specific errors here.

Reviewer #1: Yes

Reviewer #2: Yes

**********

5. Review Comments to the Author

Please use the space provided to explain your answers to the questions above. You may also include additional comments for the author, including concerns about dual publication, research ethics, or publication ethics. (Please upload your review as an attachment if it exceeds 20,000 characters)

Reviewer #1: The manuscript reads very well and highlights the results of a training program for sporting officials, an underdeveloped area in sport psychology literature. Authors managed to provide information on the quantitative and qualitative evaluation of the program, emphasising certain components.

Areas to consider for the improvement of the manuscript:

Literature review

It is informative and provides an adequate level of detail around the performed evaluation of the intervention program.

However, since the HPOP intervention contents haven't been published yet, authors need to provide more details of the actual intervention, either within the text or on Table 1. Authors need to define better the contents of workshops (i.e. conflict resolution, self-efficacy) and, the contents of the discussions. It would also be useful to clarify which components of the program were evaluated as more helpful by the participants and their trainers/mentors.

Results

In tables 2-6 it would be helpful to add vertical lines among measures and also highlight (i.e. bold fonts) the significant results among measures. These would help the reader appreciate better each measurement, changes occurring and where the significant results can be found.

Qualitative results p.16 (line 5): Authors tried to present their qualitative results within the manuscript. However, they are quite lengthy and hinder the reader's understanding of the type and details of these findings.

Authors need to run a thematic analysis based on the results of the qualitative data and present themes, subthemes and quotes accordingly. Adding a table or two would also help the reader understand better the nature of the qualitative findings.

Discussion

Since the evaluation relates to the High Performance Officiating Program (HPOP) it is important to include a section on which parts of the program, its delivery and its supporting agents were found mostly effective vs. not useful, by participants and stakeholders. This means that authors need to look at the trends in the quantitative data or examine the qualitative data with this glance. Such emphasis will highlight the potential and the strongest areas of the program, creating an important reference point and comparator for the future.

Reviewer #2: This study explored the psychosocial effects of a high-performance officiating program on five officials in Australia. There are useful insights that would be relevant to several stakeholders interested in improving the experiences of officials. Use of both quantitative and qualitative analyses yield some robust findings. Nonetheless, some weaknesses include: lack of clarity around the theoretical underpinnings of the constructs examined (why they were selected), lack of clarity around who oversees the training program for officials, commenting on unreliable (nonsignificant) quantitative results, and lack of information regarding the qualitative methods used and presentation of qualitative results. The quality of writing is generally adequate, though sentence structure is poor in some areas of the Discussion in particular. In my view this research has the potential to practically impact officiating training programs and the experiences of officials.

Introduction:

1. P3, lines 12-13: This quote about coaches being considered performers, just like their athletes, seems out of place. The sentences before it and after it emphasize the extent of research on officials, therefore a sentence (quote) strictly about coaches and athletes sandwiched in-between detracts from the flow of writing and focus on sport officials.

2. P3-4, starting line 24: Was or still is the only program for sport officials in Australia? Some clarification would be helpful as using the past tense to refer to the program but then describing the program in the present tense is a bit confusing.

3. P4, lines 10-13: Can you situate the goals of this study within the some of the relevant theories/models or previous literature on coach officiating as outlined in a recent review by Hancock and Pizzera (2025)? Saying that you will examine resilience, challenge and threat evaluation, and self-efficacy because you previously examined those psychological markers in tennis is fine, but it lacks some connection to the broader areas examined in the literature on sport officials. For instance, Hancock and Pizzera outline relevant models/ theories but also key “research areas” such as officials’ mental skills, motivations, and decision-making.

Hancock & Pizzera (2025). The psychology of sport officiating. Psychology of Sport and Exercise.

4. P5, lines 3-19: A brief yet clear explanation of what challenge and threat states/appraisals actually mean seems to be absent from this paragraph.

5. P5, line 24-25: To clarify, there are no known previous studies exploring sport officials’ experiences in training programs–in Australia or elsewhere? Or is it that these specific psychological constructs have not been examined among officials in training programs? If there has been any previous research examining officials’ experiences in training programs, it would be useful for readers to know this and for the authors to highlight this.

6. P6-7, starting line 22: The authors are investigating many factors: self-efficacy, resilience, challenge and threat appraisals, physical and mental preparation, locus of control, and functional assertiveness. Similar to my comment earlier (#2) about linking the study’s aims within a particular theory/model or key “research area/topic”, it would be helpful to articulate why these various factors are being investigated together and how they fit together from a theoretical and/or research standpoint when presenting the overarching purpose of the study. Some more emphasis on the theoretical underpinnings of this study would strengthen the manuscript.

Methods:

7. P7, line 5: Why were 6 officials initially selected? What is the justification for selecting this number of participants? It’s a small number of participants for a study, but I appreciate the applied nature of this work. Regardless, why did the program consist of 6 – and ultimately 5 – individuals?

8. P7, HPOP schedule: Is the high-performance officiating program governed/managed by a university (and this study’s authors) or a government-related sport agency? I understand earlier in the Introduction there is some mention of the program being offered by the Northern Territory Institute of Sport – but it’s not clear whether this is through a university or government sporting body. Some clarification here or in the Introduction is likely warranted and would also help readers understand why a relatively small number of officials were included in this program and why particular themes were targeted (e.g., resilience). Some additional context would be helpful to understand to what extent the authors developed this program or contributed to it.

9. P10, line 7: What were the qualitative questions for officials at time 1?

10. P10, lines 15-16: What were the qualitative questions asked to mentors about their perceptions of their officials’ resilience and self-efficacy at T2, T3, an T4? Can also remove statement about questions being the same as those from previous timepoint since qualitative questions were evidently not asked to mentors at T1.

11. P13, lines 9-10: How were the qualitative data analyzed and coded? What was the method used? I realize this section will not be as large as we would expect to see for a dedicated qualitative study, but there is no information about how the qualitative data were analyzed (e.g., inductive vs. deductive) and if (even briefly) any criteria were adopted to ensure quality standards.

Quantitative results:

12. I’m wondering whether it’s appropriate to comment on construct increases/decreases that were deemed not reliable based on the RCI. Since several constructs across the five participants did increase/decrease reliably, commenting on the unreliable increases/decreases seems questionable. Similar to traditional non-significant p-values where we would typically not make claims about increases/decreases (or relationships) that are non-significant, why comment on unreliable findings here as being meaningful? Since there are several reliable findings to draw on anyways, my suggestion would be to rely only on those (which puts more confidence in the findings and enhances the quality of the research and associated claims).

Qualitative results:

13. P16, resilience: Jade’s quotes regarding perceptions of resilience are repeated verbatim at different time points – this seems unlikely.

14. P18, line 13: This paragraph seems like you are introducing the qualitative findings on self-efficacy for the first time here. However, this is the third paragraph about this finding that was introduced on P17, and it is repetitive. Overall, I would encourage the authors to review their qualitative findings and look for areas to enhance the flow of writing – ensuring results are succint and not repeated.

Discussion:

15. P22, 11-18: Since there is not much emphasis in the paper on how the mentor and mentee interacted during the program (e.g., how often, topics discussed), this paragraph could be a good opportunity to relate the findings to previous research that has pointed to the value of mentee-mentor relationships among officials. For instance, you could point to previous research that highlighted the important role of senior officials in mentoring younger officials in ice hockey (Hancock et al., 2018).

Hancock, D. J., Martin, L. J., Evans, M. B., & Paradis, K. F. (2018). Exploring Perceptions of Group Processes in Ice Hockey Officiating. Journal of Applied Sport Psychology, 30(2), 222-240. https://doi.org/10.1080/10413200.2017.1349208

Minor comments:

16. No need for period after title.

17. Headings do not follow PLOS One formatting guidelines.

18. P4, line 16: “Positively”?

19. P6, line 2: “Importance”?

20. P6, lines 7-9: Awkward sentence structure. Please re-phrase.

21. P9, line 23: Obtaining ethical approval was already mentioned earlier in the Methods. Pick one place to make this statement.

22. P9, line 24: Data were collected.

23. P10, line: Complete “it”? Something is missing from this sentence.

24. P16, line 6: “to” what

25. P16, lines 20-24: Abrupt transition to Alex’s mentor’s views at Time 4, then moving to Alex’s (official) views at time 2. This seems out of order and a bit hard to follow.

26. P16, line 25: “This is reinforced by Meg (at time 2) stated…” Sentence structure error here.

27. P20, lines 23-25: This is an incomplete sentence.

28. P21, lines 4-7: Long, choppy sentence. Please revise.

29. P21, lines 15-19: Long, choppy sentence. Please revise.

30. P21, 19-20: Unclear what this sentence means.

31. P22, lines 11-13: A comma is needed after “relationship.”

**********

6. PLOS authors have the option to publish the peer review history of their article (what does this mean?). If published, this will include your full peer review and any attached files.

If you choose “no”, your identity will remain anonymous but your review may still be made public.

**Do you want your identity to be public for this peer review?**  For information about this choice, including consent withdrawal, please see our Privacy Policy.

Reviewer #1: **Yes:**  Dr Emmanouil (Manos) Georgiadis

Reviewer #2: No

**********

[NOTE: If reviewer comments were submitted as an attachment file, they will be attached to this email and accessible via the submission site. Please log into your account, locate the manuscript record, and check for the action link "View Attachments". If this link does not appear, there are no attachment files.]

To ensure your figures meet our technical requirements, please review our figure guidelines: https://journals.plos.org/plosone/s/figures

You may also use PLOS’s free figure tool, NAAS, to help you prepare publication quality figures: https://journals.plos.org/plosone/s/figures#loc-tools-for-figure-preparation.

NAAS will assess whether your figures meet our technical requirements by comparing each figure against our figure specifications.

# Author comment: Author response to Decision Letter 1

**DOI:** 10.1371/journal.pone.0354971.r002

24 May 2026

Responses to Reviews

Manuscript No. PONE-D-25-62568

Evaluating the psychosocial effects of the Northern Territory Institute of Sport (NTIS) High Performance Officiating Program (HPOP)

PLOS ONE

Firstly, let us thank the Co-Editors and the reviewers for taking the time to review the manuscript and for providing thoughtful and constructive comments. We have endeavoured to address each comment and have shown the changes to the manuscript in red text.

Reviewer #1 comments:

The manuscript reads very well and highlights the results of a training program for sporting officials, an underdeveloped area in sport psychology literature. Authors managed to provide information on the quantitative and qualitative evaluation of the program, emphasising certain components.

We thank the reviewer #1 for taking the time to provide very detailed comments on our manuscript and we have endeavoured to address all of their points.

Specific comments

Literature Review

It is informative and provides an adequate level of detail around the performed evaluation of the intervention program.

However, since the HPOP intervention contents haven't been published yet, authors need to provide more details of the actual intervention, either within the text or on Table 1. Authors need to define better the contents of workshops (i.e. conflict resolution, self-efficacy) and, the contents of the discussions. It would also be useful to clarify which components of the program were evaluated as more helpful by the participants and their trainers/mentors.

In response, we have substantially expanded the description of the HPOP. Table 1 (page 34) now provides a detailed overview of session content, including workshop topics (e.g., resilience, self efficacy, conflict resolution, pressure management) and the focus of facilitated discussions. Additionally, clarification has also been added within the text to highlight program components that were perceived as particularly valuable based on participant and mentor feedback. For example, the increased opportunity to officiate (page 16, lines 7-9), and physical and mental preparation (Page 19, lines 8-10),

Results

In tables 2-6 it would be helpful to add vertical lines among measures and also highlight (i.e. bold fonts) the significant results among measures. These would help the reader appreciate better each measurement, changes occurring and where the significant results can be found.

We have revised Tables 2–6 accordingly. Vertical lines have been added to clearly separate constructs, and statistically reliable changes (based on the Reliable Change Index criterion) are now highlighted using bold formatting to aid readability.

Qualitative results p.16 (line 5): Authors tried to present their qualitative results within the manuscript. However, they are quite lengthy and hinder the reader's understanding of the type and details of these findings.

Authors need to run a thematic analysis based on the results of the qualitative data and present themes, subthemes and quotes accordingly. Adding a table or two would also help the reader understand better the nature of the qualitative findings.

Thank you for the suggestion. We thought about the discussed the reviewer’s comment a lot, and decided to bring onto the project a colleague to undertake a more substantial and meaningful analysis of the qualitative data. This allowed the data to be view more objectively and has added more value to the paper. Thus, we have conducted a thematic analysis and presented both higher and lower themes through a Figure. Appropriate quotes have been used, and we have also added two tables with representative quotes of the lower order themes, whilst being careful not to add too much more text to the paper.

Discussion

Since the evaluation relates to the High Performance Officiating Program (HPOP) it is important to include a section on which parts of the program, its delivery and its supporting agents were found mostly effective vs. not useful, by participants and stakeholders. This means that authors need to look at the trends in the quantitative data or examine the qualitative data with this glance. Such emphasis will highlight the potential and the strongest areas of the program, creating an important reference point and comparator for the future.

Based on the reviewers comments we have discussed the strengths of the program through the thematic analysis. For example, Figure 1 (page 44), shows that social support was an important lower order factor, likewise, quotes such as “I have also gained from all the mentors and other officials in the program” (page 42, table 9). All of the lower-order factors have been identified in the paper, through the results, discussion, tables (9-10) and Figure 1.

Reviewer #2 Comments

This study explored the psychosocial effects of a high-performance officiating program on five officials in Australia. There are useful insights that would be relevant to several stakeholders interested in improving the experiences of officials. Use of both quantitative and qualitative analyses yield some robust findings. Nonetheless, some weaknesses include: lack of clarity around the theoretical underpinnings of the constructs examined (why they were selected), lack of clarity around who oversees the training program for officials, commenting on unreliable (nonsignificant) quantitative results, and lack of information regarding the qualitative methods used and presentation of qualitative results. The quality of writing is generally adequate, though sentence structure is poor in some areas of the Discussion in particular. In my view this research has the potential to practically impact officiating training programs and the experiences of officials.

We thank the reviewer #2 for taking the time to provide very detailed comments on our manuscript and we have endeavoured to address all of their points to improve theoretical grounding, methodological clarity and overall readability of the manuscript.

Specific comments

Introduction:

1. P3, lines 12-13: This quote about coaches being considered performers, just like their athletes, seems out of place. The sentences before it and after it emphasize the extent of research on officials, therefore a sentence (quote) strictly about coaches and athletes sandwiched in-between detracts from the flow of writing and focus on sport officials.

P2, lines 13-15. Thank you for the feedback. The quoted sentence has been removed, and the surrounding text has been revised to maintain focus on sport officials. Reference numbering has been updated accordingly.

2. P3-4, starting line 24: Was or still is the only program for sport officials in Australia? Some clarification would be helpful as using the past tense to refer to the program but then describing the program in the present tense is a bit confusing.

P2-3, starting line 23. The relevant section has been revised to clarify that the HPOP represents the only high-performance officiating program catering for a range of sporting officials at the time. Tense usage has been corrected for consistency (page 3, lines 2-4).

3. P4, lines 10-13: Can you situate the goals of this study within the some of the relevant theories/models or previous literature on coach officiating as outlined in a recent review by Hancock and Pizzera (2025)? Saying that you will examine resilience, challenge and threat evaluation, and self-efficacy because you previously examined those psychological markers in tennis is fine, but it lacks some connection to the broader areas examined in the literature on sport officials. For instance, Hancock and Pizzera outline relevant models/ theories but also key “research areas” such as officials’ mental skills, motivations, and decision-making.

Hancock & Pizzera (2025). The psychology of sport officiating. Psychology of Sport and Exercise.

Page 6, Lines 1-18. Thank you for providing the reference. We have strengthened the theoretical framework by explicitly positioning the study within the Theory of Challenge and Threat States in Athletes (TCTSA). This now clearly explains how the selected constructs align with key theoretical and applied research.

4. P5, lines 3-19: A brief yet clear explanation of what challenge and threat states/appraisals actually mean seems to be absent from this paragraph.

Page 4, lines 4-7. A concise explanation of challenge and threat appraisals has been added to the introduction to ensure conceptual clarity for readers.

5. P5, line 24-25: To clarify, there are no known previous studies exploring sport officials’ experiences in training programs–in Australia or elsewhere? Or is it that these specific psychological constructs have not been examined among officials in training programs? If there has been any previous research examining officials’ experiences in training programs, it would be useful for readers to know this and for the authors to highlight this.

Page 6, lines 19-20. We now explicitly state that this study represents the first empirical evaluation of the HPOP, addressing this point directly.

6. P6-7, starting line 22: The authors are investigating many factors: self-efficacy, resilience, challenge and threat appraisals, physical and mental preparation, locus of control, and functional assertiveness. Similar to my comment earlier (#2) about linking the study’s aims within a particular theory/model or key “research area/topic”, it would be helpful to articulate why these various factors are being investigated together and how they fit together from a theoretical and/or research standpoint when presenting the overarching purpose of the study. Some more emphasis on the theoretical underpinnings of this study would strengthen the manuscript.

Page 6, lines 1-18. Thank you for the feedback. We have now clearly articulated how the study’s constructs collectively aligns with the TCTSA framework, including previous research.

Methods:

7. P7, line 5: Why were 6 officials initially selected? What is the justification for selecting this number of participants? It’s a small number of participants for a study, but I appreciate the applied nature of this work. Regardless, why did the program consist of 6 – and ultimately 5 – individuals?

Page 7, lines 1-2. All officials who applied for the HPOP were included in the study. An explanation has been provided regarding the selection of the officials.

8. P7, HPOP schedule: Is the high-performance officiating program governed/managed by a university (and this study’s authors) or a government-related sport agency? I understand earlier in the Introduction there is some mention of the program being offered by the Northern Territory Institute of Sport – but it’s not clear whether this is through a university or government sporting body. Some clarification here or in the Introduction is likely warranted and would also help readers understand why a relatively small number of officials were included in this program and why particular themes were targeted (e.g., resilience). Some additional context would be helpful to understand to what extent the authors developed this program or contributed to it.

Pages 7-8, starts on line 22. The methods section now clarifies that the HPOP was designed and delivered by the Northern Territory Institute of Sport (NTIS), with the research team evaluating the program rather than governing it.

9. P10, line 7: What were the qualitative questions for officials at time 1?

Page 10, lines 7-16. No qualitative questions were asked at Time 1. This has been fixed. All qualitative questions asked of the officials and mentors at later time points have been explicitly listed and redundant statements have been removed.

10. P10, lines 15-16: What were the qualitative questions asked to mentors about their perceptions of their officials’ resilience and self-efficacy at T2, T3, an T4? Can also remove statement about questions being the same as those from previous timepoint since qualitative questions were evidently not asked to mentors at T1.

Page 10, lines 17-23. The qualitative questions have been added, likewise the statement has been removed.

11. P13, lines 9-10: How were the qualitative data analyzed and coded? What was the method used? I realize this section will not be as large as we would expect to see for a dedicated qualitative study, but there is no information about how the qualitative data were analyzed (e.g., inductive vs. deductive) and if (even briefly) any criteria were adopted to ensure quality standards.

Page 13-14. Starts on line 17. Thank you for the feedback, we have undertaken a thematic analysis (as per reviewer 1’s comments) with detailed information on how it was undertaken in this section.

Quantitative results:

12. I’m wondering whether it’s appropriate to comment on construct increases/decreases that were deemed not reliable based on the RCI. Since several constructs across the five participants did increase/decrease reliably, commenting on the unreliable increases/decreases seems questionable. Similar to traditional non-significant p-values where we would typically not make claims about increases/decreases (or relationships) that are non-significant, why comment on unreliable findings here as being meaningful? Since there are several reliable findings to draw on anyways, my suggestion would be to rely only on those (which puts more confidence in the findings and enhances the quality of the research and associated claims).

Pages14-15, starts on line 7. All non-reliable changes have now been removed. The results and discussion sections focus exclusively on reliable RCI findings to strengthen the validity of claims.

Qualitative results:

13. P16, resilience: Jade’s quotes regarding perceptions of resilience are repeated verbatim at different time points – this seems unlikely.

Due to the changes made in regard to the qualitative analysis, Jade’s comments have been removed as they no longer fit within the article.

14. P18, line 13: This paragraph seems like you are introducing the qualitative findings on self-efficacy for the first time here. However, this is the third paragraph about this finding that was introduced on P17, and it is repetitive. Overall, I would encourage the authors to review their qualitative findings and look for areas to enhance the flow of writing – ensuring results are succint and not repeated.

As noted previously we have reviewed the qualitative findings to enhance the flow of the writing.

Discussion:

15. P22, 11-18: Since there is not much emphasis in the paper on how the mentor and mentee interacted during the program (e.g., how often, topics discussed), this paragraph could be a good opportunity to relate the findings to previous research that has pointed to the value of mentee-mentor relationships among officials. For instance, you could point to previous research that highlighted the important role of senior officials in mentoring younger officials in ice hockey (Hancock et al., 2018).

Page 21, lines 22-25. Thank you for the reference, the discussion now explicitly links findings to prior mentoring research in officiating, including work by Hancock et al (2018), highlighting the role of mentor-mentee relationship.

Hancock, D. J., Martin, L. J., Evans, M. B., & Paradis, K. F. (2018). Exploring Perceptions of Group Processes in Ice Hockey Officiating. Journal of Applied Sport Psychology, 30(2), 222-240. https://doi.org/10.1080/10413200.2017.1349208

Minor comments:

16. No need for period after title.

17. Headings do not follow PLOS One formatting guidelines.

18. P4, line 16: “Positively”?

19. P6, line 2: “Importance”?

20. P6, lines 7-9: Awkward sentence structure. Please re-phrase.

21. P9, line 23: Obtaining ethical approval was already mentioned earlier in the Methods. Pick one place to make this statement.

22. P9, line 24: Data were collected.

23. P10, line: Complete “it”? Something is missing from this sentence.

24. P16, line 6: “to” what

25. P16, lines 20-24: Abrupt transition to Alex’s mentor’s views at Time 4, then moving to Alex’s (official) views at time 2. This seems out of order and a bit hard to follow.

26. P16, line 25: “This is reinforced by Meg (at time 2) stated…” Sentence

Attachment Submitted filename: Responses to Reviewers Comments HPOP May 2026.docx

# Peer review: Decision Letter 1

**DOI:** 10.1371/journal.pone.0354971.r003

**Authors:** Ender Senel

9 Jul 2026

PONE-D-25-62568R1

Evaluating the psychosocial effects of the Northern Territory Institute of Sport (NTIS) High Performance Officiating Program (HPOP).

PLOS One

Dear Dr. Turner,

Thank you for submitting your manuscript to PLOS ONE. After careful consideration, we feel that it has merit but does not fully meet PLOS ONE’s publication criteria as it currently stands. Therefore, we invite you to submit a revised version of the manuscript that addresses the points raised during the review process.

Please submit your revised manuscript by Aug 23 2026 11:59PM. If you will need more time than this to complete your revisions, please reply to this message or contact the journal office at plosone@plos.org. When you're ready to submit your revision, log on to https://www.editorialmanager.com/pone/ and select the 'Submissions Needing Revision' folder to locate your manuscript file.

Please include the following items when submitting your revised manuscript:

- A letter that responds to each point raised by the academic editor and reviewer(s). You should upload this letter as a separate file labeled 'Response to Reviewers'.
- A marked-up copy of your manuscript that highlights changes made to the original version. You should upload this as a separate file labeled 'Revised Manuscript with Track Changes'.
- An unmarked version of your revised paper without tracked changes. You should upload this as a separate file labeled 'Manuscript'.

If you would like to make changes to your financial disclosure, please include your updated statement in your cover letter. Guidelines for resubmitting your figure files are available below the reviewer comments at the end of this letter.

If applicable, we recommend that you deposit your laboratory protocols in protocols.io to enhance the reproducibility of your results. Protocols.io assigns your protocol its own identifier (DOI) so that it can be cited independently in the future. For instructions see: https://journals.plos.org/plosone/s/submission-guidelines#loc-laboratory-protocols. Additionally, PLOS ONE offers an option for publishing peer-reviewed Lab Protocol articles, which describe protocols hosted on protocols.io. Read more information on sharing protocols at https://plos.org/protocols?utm_medium=editorial-email&utm_source=authorletters&utm_campaign=protocols.

As the corresponding author, your ORCID iD is verified in the submission system and will appear in the published article. PLOS supports the use of ORCID, and we encourage all coauthors to register for an ORCID iD and use it as well. Please encourage your coauthors to verify their ORCID iD within the submission system before final acceptance, as unverified ORCID iDs will not appear in the published article. *Only*  the individual author can complete the verification step; PLOS staff *cannot*  verify ORCID iDs on behalf of authors.

We look forward to receiving your revised manuscript.

Kind regards,

Ender Senel, PhD

Academic Editor

PLOS One

**Journal Requirements:**

1. If the reviewer comments include a recommendation to cite specific previously published works, please review and evaluate these publications to determine whether they are relevant and should be cited. There is no requirement to cite these works unless the editor has indicated otherwise.

2. Please review your reference list to ensure that it is complete and correct. If you have cited papers that have been retracted, please include the rationale for doing so in the manuscript text, or remove these references and replace them with relevant current references. Any changes to the reference list should be mentioned in the rebuttal letter that accompanies your revised manuscript. If you need to cite a retracted article, indicate the article’s retracted status in the References list and also include a citation and full reference for the retraction notice.

[Note: HTML markup is below. Please do not edit.]

Reviewers' comments:

Reviewer's Responses to Questions

**Comments to the Author**

1. If the authors have adequately addressed your comments raised in a previous round of review and you feel that this manuscript is now acceptable for publication, you may indicate that here to bypass the “Comments to the Author” section, enter your conflict of interest statement in the “Confidential to Editor” section, and submit your "Accept" recommendation.

Reviewer #2: (No Response)

**********

2. Is the manuscript technically sound, and do the data support the conclusions?

The manuscript must describe a technically sound piece of scientific research with data that supports the conclusions. Experiments must have been conducted rigorously, with appropriate controls, replication, and sample sizes. The conclusions must be drawn appropriately based on the data presented.

Reviewer #2: Partly

**********

3. Has the statistical analysis been performed appropriately and rigorously?

Reviewer #2: Yes

**********

4. Have the authors made all data underlying the findings in their manuscript fully available?

The PLOS Data policy requires authors to make all data underlying the findings described in their manuscript fully available without restriction, with rare exception (please refer to the Data Availability Statement in the manuscript PDF file). The data should be provided as part of the manuscript or its supporting information, or deposited to a public repository. For example, in addition to summary statistics, the data points behind means, medians and variance measures should be available. If there are restrictions on publicly sharing data—e.g. participant privacy or use of data from a third party—those must be specified.

Reviewer #2: Yes

**********

5. Is the manuscript presented in an intelligible fashion and written in standard English?

PLOS ONE does not copyedit accepted manuscripts, so the language in submitted articles must be clear, correct, and unambiguous. Any typographical or grammatical errors should be corrected at revision, so please note any specific errors here.

Reviewer #2: Yes

**********

6. Review Comments to the Author

Please use the space provided to explain your answers to the questions above. You may also include additional comments for the author, including concerns about dual publication, research ethics, or publication ethics. (Please upload your review as an attachment if it exceeds 20,000 characters)

**Reviewer #2:**  I appreciate the revisions made by the authors on this revised manuscript. The quality of the manuscript has improved. Nonetheless, some areas of concern remain. Please see my comments below.

1. Abstract: Please review the abstract to ensure it aligns with the results that are presented. There is language around “…increases/improvements, but only XX participants had reliable increases.” This is confusing and does not align with the results, which the authors noted now only focus on significant/reliable changes.

2. P2, line 23: Suggest changing “However” to “Further”, since emphasizing that only one program existed for a twelve-month period at the time aligns with the previous sentence about there being few (limited) training programs for officials. Currently it reads like a contrast, but that seems incorrect.

Also suggest inserting the year(s) when this program occurred here, since it’s not clear to the reader at this point in the paper when this 12-month period occurred.

3. P6, line 6: I think “psychological” resilience here.

4. P6, line 11: Suggest comma after TCTSA.

5. P6, lines 14-18: Revise, some issues with the sentence structure.

6. P10, qualitative questions: Why do the questions for both officials and mentors imply that official self-efficacy and resilience must have increased? (e.g., Please give an example of how you have shown increased self-efficacy since commencing in the HPOP?). This choice of wording would certainly have the potential to bias the results.

7. P13-14, qualitative data analysis: The six stages of data analysis are poorly written. Please revise.

8. P14-15, quantitative results: I’m a bit confused with some of the subscales/factors that show up in the results and the tables (e.g., functional assertiveness subscales) but don’t appear to be clearly highlighted as distinct subscales earlier in the measures section. Please ensure all subscales that are mentioned in the results and tables are clearly identified within the measures section (so that the reader knows in advance exactly what subscales to expect later in the results).

9. P15-16, qualitative results: I realize there is a desire to keep the qualitative section shorter, but I find the results rather thin on detail and context. In particular, the lower-order themes are not actually described. Rather, they are merely stated with a representative quote. At minimum, there needs to be a brief description of what these themes mean (ie., define the themes).

10. P16, lines 7-9: Incomplete sentence regarding lower-order themes.

11. P20, line 12: Period in middle of sentence.

**********

7. PLOS authors have the option to publish the peer review history of their article (what does this mean?). If published, this will include your full peer review and any attached files.

If you choose “no”, your identity will remain anonymous but your review may still be made public.

**Do you want your identity to be public for this peer review?**  For information about this choice, including consent withdrawal, please see our Privacy Policy.

Reviewer #2: No

**********

[NOTE: If reviewer comments were submitted as an attachment file, they will be attached to this email and accessible via the submission site. Please log into your account, locate the manuscript record, and check for the action link "View Attachments". If this link does not appear, there are no attachment files.]

To ensure your figures meet our technical requirements, please review our figure guidelines: https://journals.plos.org/plosone/s/figures

You may also use PLOS’s free figure tool, NAAS, to help you prepare publication quality figures: https://journals.plos.org/plosone/s/figures#loc-tools-for-figure-preparation.

NAAS will assess whether your figures meet our technical requirements by comparing each figure against our figure specifications.

# Author comment: Author response to Decision Letter 2

**DOI:** 10.1371/journal.pone.0354971.r004

14 Jul 2026

Responses to Reviews

Manuscript No. PONE-D-25-62568

Evaluating the psychosocial effects of the Northern Territory Institute of Sport (NTIS) High Performance Officiating Program (HPOP)

PLOS ONE

Firstly, let us thank the Co-Editors and the reviewers for taking the time to review the manuscript and for providing thoughtful and constructive comments. We have endeavoured to address each comment and have shown the changes to the manuscript in red text.

Reviewer #2 comments:

Abstract: Please review the abstract to ensure it aligns with the results that are presented. There is language around “…increases/improvements, but only XX participants had reliable increases.” This is confusing and does not align with the results, which the authors noted now only focus on significant/reliable changes.

Thank you for this suggestion. We agree that the previous wording could be interpreted as implying improvements across all participants. The abstract has been revised to focus on reliable improvements identified through the Reliable Change Indices (RCIs), with self-efficacy and resilience findings now reported in a manner consistent with the results section. Additional wording was included to acknowledge the variability of responses across individual officials.

2. P2, line 23: Suggest changing “However” to “Further”, since emphasizing that only one program existed for a twelve-month period at the time aligns with the previous sentence about there being few (limited) training programs for officials. Currently it reads like a contrast, but that seems incorrect.

Page 2, line 23. Changed “However” to “Further”.

Also suggest inserting the year(s) when this program occurred here, since it’s not clear to the reader at this point in the paper when this 12-month period occurred.

Page 2, line 23. We have added the year (2021) at the first mention of the NTIS High Performance Officiating Program (HPOP).

3. P6, line 6: I think “psychological” resilience here.

Page 6, line 6. We have changed “Psychology” to “Psychological”.

4. P6, line 11: Suggest comma after TCTSA.

Page 6, line 11, have added a comma after TCTSA.

5. P6, lines 14-18: Revise, some issues with the sentence structure.

Page 6, lines 15-18. Thank you for highlighting this issue. The sentence has been revised to improve clarity, grammar, and flow to articulate the relevance of challenge-threat theory to non-athlete performance settings, including sports officials.

6. P10, qualitative questions: Why do the questions for both officials and mentors imply that official self-efficacy and resilience must have increased? (e.g., Please give an example of how you have shown increased self-efficacy since commencing in the HPOP?). This choice of wording would certainly have the potential to bias the results.

Thank you for raising this important point. We acknowledge that the wording of some interview questions may have encouraged participants and mentors to reflect specifically on positive changes in self-efficacy and resilience. The interviews were intentionally focused on exploring these constructs, as they were key target outcomes of the HPOP, however, we recognise that the phrasing may have introduced a degree of positive-response bias and may have limited opportunities for participants to discuss no change or negative experiences. This limitation has now been acknowledged in the discussion section (Page 23, lines 17-24), and we recommend that future research employ more neutral interview questions to allow for a broader range of participant responses.

7. P13-14, qualitative data analysis: The six stages of data analysis are poorly written. Please revise.

We have revised this section as requested and, while the original writing represented how the stages were presented by Nowell et al. (2017), we have written the stages in a more continuous prose to promote fluency. (Page 15, lines 2-10).

8. P14-15, quantitative results: I’m a bit confused with some of the subscales/factors that show up in the results and the tables (e.g., functional assertiveness subscales) but don’t appear to be clearly highlighted as distinct subscales earlier in the measures section. Please ensure all subscales that are mentioned in the results and tables are clearly identified within the measures section (so that the reader knows in advance exactly what subscales to expect later in the results).

Page 13, lines 3-7. Thank you for this observation. We have revised the method section to clearly identify and describe the two subscales as reported in the results and tables.

9. P15-16, qualitative results: I realize there is a desire to keep the qualitative section shorter, but I find the results rather thin on detail and context. In particular, the lower-order themes are not actually described. Rather, they are merely stated with a representative quote. At minimum, there needs to be a brief description of what these themes mean (ie., define the themes).

Thank you for this recommendation that, we believe, has enhanced the detail of the qualitative results. Amendments have been made on page 17 (lines 5-11 and lines 13-18) where definitions of the lower order and higher order themes are defined.

10. P16, lines 7-9: Incomplete sentence regarding lower-order themes.

Thank you for highlighting this issue. The incomplete sentence as been corrected and revised to clearly describe the lower-order themes identified within the qualitative analysis.

11. P20, line 12: Period in middle of sentence.

Fixed as requested

Attachment Submitted filename: Responses to Reviewers.docx

# Editor report: Decision Letter 2

**DOI:** 10.1371/journal.pone.0354971.r005

**Authors:** Ender Senel

15 Jul 2026

Evaluating the psychosocial effects of the Northern Territory Institute of Sport (NTIS) High Performance Officiating Program (HPOP).

PONE-D-25-62568R2

Dear Dr. Turner,

We’re pleased to inform you that your manuscript has been judged scientifically suitable for publication and will be formally accepted for publication once it meets all outstanding technical requirements.

Within one week, you’ll receive an e-mail detailing the required amendments. When these have been addressed, you’ll receive a formal acceptance letter and your manuscript will be scheduled for publication.

An invoice will be generated when your article is formally accepted. Please note, if your institution has a publishing partnership with PLOS and your article meets the relevant criteria, all or part of your publication costs will be covered. Please make sure your user information is up-to-date by logging into Editorial Manager at Editorial Manager® and clicking the ‘Update My Information' link at the top of the page. For questions related to billing, please contact billing support.

If your institution or institutions have a press office, please notify them about your upcoming paper to help maximize its impact. If they’ll be preparing press materials, please inform our press team as soon as possible -- no later than 48 hours after receiving the formal acceptance. Your manuscript will remain under strict press embargo until 2 pm Eastern Time on the date of publication. For more information, please contact onepress@plos.org.

Kind regards,

Ender Senel, PhD

Academic Editor

PLOS One

Additional Editor Comments (optional):

Reviewers' comments:

# Editor report: Acceptance letter

**DOI:** 10.1371/journal.pone.0354971.r006

**Authors:** Ender Senel

PONE-D-25-62568R2

PLOS One

Dear Dr. Turner,

I'm pleased to inform you that your manuscript has been deemed suitable for publication in PLOS One. Congratulations! Your manuscript is now being handed over to our production team.

At this stage, our production department will prepare your paper for publication. This includes ensuring the following:

* All references, tables, and figures are properly cited

* All relevant supporting information is included in the manuscript submission,

* There are no issues that prevent the paper from being properly typeset

You will receive further instructions from the production team, including instructions on how to review your proof when it is ready. Please keep in mind that we are working through a large volume of accepted articles, so please give us a few days to review your paper and let you know the next and final steps.

Lastly, if your institution or institutions have a press office, please let them know about your upcoming paper now to help maximize its impact. If they'll be preparing press materials, please inform our press team within the next 48 hours. Your manuscript will remain under strict press embargo until 2 pm Eastern Time on the date of publication. For more information, please contact onepress@plos.org.

You will receive an invoice from PLOS for your publication fee after your manuscript has reached the completed accept phase. If you receive an email requesting payment before acceptance or for any other service, this may be a phishing scheme. Learn how to identify phishing emails and protect your accounts at https://explore.plos.org/phishing.

If we can help with anything else, please email us at customercare@plos.org.

Thank you for submitting your work to PLOS ONE and supporting open access.

Kind regards,

PLOS ONE Editorial Office Staff

on behalf of

Dr. Ender Senel

Academic Editor

PLOS One
